# Effects of Testing and Disclosing Ancestry-Specific Genetic Risk for Kidney Failure on Patients and Health Care Professionals: A Randomized Clinical Trial

**DOI:** 10.1001/jamanetworkopen.2022.1048

**Published:** 2022-03-04

**Authors:** Girish N. Nadkarni, Kezhen Fei, Michelle A. Ramos, Diane Hauser, Emilia Bagiella, Stephen B. Ellis, Saskia Sanderson, Stuart A. Scott, Tatiana Sabin, Ebony Madden, Richard Cooper, Martin Pollak, Neil Calman, Erwin P. Bottinger, Carol R. Horowitz

**Affiliations:** 1 Division of Nephrology, Department of Medicine, Icahn School of Medicine at Mount Sinai, New York, New York; 2 The Charles Bronfman Institute for Personalized Medicine, Icahn School of Medicine at Mount Sinai, New York, New York; 3 Department of Population Health Sciences and Policy, Icahn School of Medicine at Mount Sinai, New York, New York; 4 Institute for Health Equity Research, Icahn School of Medicine at Mount Sinai, New York, New York; 5 Institute for Family Health, New York, New York; 6 Department of Genetics and Genomic Sciences, Icahn School of Medicine at Mount Sinai, New York, New York; 7 Sema4, A Mount Sinai Venture, Stamford, Connecticut; 8 National Human Genome Research Institute, Bethesda, Maryland; 9 Department of Public Health Sciences, Loyola University Medical School, Maywood, Illinois; 10 Division of Nephrology, Harvard Medical School, Boston, Massachusetts; 11 Digital Health Center, Hasso Plattner Institute, Potsdam, Germany

## Abstract

**Importance:**

Risk variants in the apolipoprotein L1 (*APOL1* [OMIM 603743]) gene on chromosome 22 are common in individuals of West African ancestry and confer increased risk of kidney failure for people with African ancestry and hypertension. Whether disclosing *APOL1* genetic testing results to patients of African ancestry and their clinicians affects blood pressure, kidney disease screening, or patient behaviors is unknown.

**Objective:**

To determine the effects of testing and disclosing *APOL1* genetic results to patients of African ancestry with hypertension and their clinicians.

**Design, Setting, and Participants:**

This pragmatic randomized clinical trial randomly assigned 2050 adults of African ancestry with hypertension and without existing chronic kidney disease in 2 US health care systems from November 1, 2014, through November 28, 2016; the final date of follow-up was January 16, 2018. Patients were randomly assigned to undergo immediate (intervention) or delayed (waiting list control group) *APOL1* testing in a 7:1 ratio. Statistical analysis was performed from May 1, 2018, to July 31, 2020.

**Interventions:**

Patients randomly assigned to the intervention group received *APOL1* genetic testing results from trained staff; their clinicians received results through clinical decision support in electronic health records. Waiting list control patients received the results after their 12-month follow-up visit.

**Main Outcomes and Measures:**

Coprimary outcomes were the change in 3-month systolic blood pressure and 12-month urine kidney disease screening comparing intervention patients with high-risk *APOL1* genotypes and those with low-risk *APOL1* genotypes. Secondary outcomes compared these outcomes between intervention group patients with high-risk *APOL1* genotypes and controls. Exploratory analyses included psychobehavioral factors.

**Results:**

Among 2050 randomly assigned patients (1360 women [66%]; mean [SD] age, 53 [10] years), the baseline mean (SD) systolic blood pressure was significantly higher in patients with high-risk *APOL1* genotypes vs those with low-risk *APOL1* genotypes and controls (137 [21] vs 134 [19] vs 133 [19] mm Hg; *P* = .003 for high-risk vs low-risk *APOL1* genotypes; *P* = .001 for high-risk *APOL1* genotypes vs controls). At 3 months, the mean (SD) change in systolic blood pressure was significantly greater in patients with high-risk *APOL1* genotypes vs those with low-risk *APOL1* genotypes (6 [18] vs 3 [18] mm Hg; *P* = .004) and controls (6 [18] vs 3 [19] mm Hg; *P* = .01). At 12 months, there was a 12% increase in urine kidney disease testing among patients with high-risk *APOL1* genotypes (from 39 of 234 [17%] to 68 of 234 [29%]) vs a 6% increase among those with low-risk *APOL1* genotypes (from 278 of 1561 [18%] to 377 of 1561 [24%]; *P* = .10) and a 7% increase among controls (from 33 of 255 [13%] to 50 of 255 [20%]; *P* = .01). In response to testing, patients with high-risk *APOL1* genotypes reported more changes in lifestyle (a subjective measure that included better dietary and exercise habits; 129 of 218 [59%] vs 547 of 1468 [37%]; *P* < .001) and increased blood pressure medication use (21 of 218 [10%] vs 68 of 1468 [5%]; *P* = .005) vs those with low-risk *APOL1* genotypes; 1631 of 1686 (97%) declared they would get tested again.

**Conclusions and Relevance:**

In this randomized clinical trial, disclosing *APOL1* genetic testing results to patients of African ancestry with hypertension and their clinicians was associated with a greater reduction in systolic blood pressure, increased kidney disease screening, and positive self-reported behavior changes in those with high-risk genotypes.

**Trial Registration:**

ClinicalTrials.gov Identifier: NCT02234063

## Key Points

**Question:**

What are the effects of testing and disclosing the genetic results for *APOL1* on patients of African ancestry with hypertension and their clinicians?

**Findings:**

In this randomized clinical trial of 2050 patients of African ancestry with hypertension without chronic kidney disease in which genetic testing results were disclosed to patients and clinicians, patients with high-risk *APOL1* genotypes had greater improvement in blood pressure from baseline and more lifestyle changes (better dietary and exercise habits) compared with patients with low-risk *APOL1* genotypes or waiting list control patients.

**Meaning:**

Disclosing *APOL1* genetic testing results to patients of African ancestry with hypertension and to their clinicians was associated with a greater reduction in blood pressure, kidney disease screening, and self-reported behavior changes in those with high-risk *APOL1* genotypes.

## Teaser

This randomized clinical trial determines the effects of testing and disclosing *APOL1* genetic results on patients of African ancestry with hypertension and their clinicians.

## Introduction

Chronic kidney disease (CKD) affects 26 million US adults.^1^ Individuals of African ancestry have a higher risk of CKD and end-stage kidney disease than individuals with European ancestry owing to social determinants, clinical factors, and health system factors.^1,2,3^ Race and ethnicity are social constructs, but ancestry has some biological underpinnings. High-risk genotypes at the apolipoprotein L1 (APOL1) locus confer a 5-fold to 10-fold increased risk for CKD and end-stage kidney disease attributed to hypertension, although this risk increment is attenuated among individuals with diabetes.^4,5,6^ High-risk variants of *APOL1* (OMIM 603743) on chromosome 22 are found in 1 of 7 people of African ancestry but are nearly absent in people of European ancestry^7^ because they confer resistance to trypanosomal infection and are subject to positive selection in West Africa.^8,9^

Although it is important to avoid overstating the importance of genetic contributions to chronic disease disparities and to acknowledge that social determinants of health are the critical determinants of disparities,^10^ racial and ethnic minority populations should not be the last to benefit from scientific advances, including genetic discoveries. There is increasing interest in incorporating genetic testing into primary care.^11,12,13^ The *APOL1* risk genotype is common and confers high risk for a serious chronic disease in people of African ancestry, who are disproportionately burdened by chronic diseases, and thus it may be important to incorporate genetic testing into clinical care.^11,12^ Blood pressure (BP) control reduces kidney function deterioration,^14^ but people of African ancestry have the highest age-adjusted prevalence of hypertension and the lowest rates of blood pressure control.^15^ Kidney function tests, including urine microalbumin testing, aid in risk stratification and clinical staging but are underused among patients at high risk of CKD, especially patients of African ancestry.^16^

Stricter BP control may have a mortality benefit for persons with high-risk *APOL1* genotypes,^17^ but little is known about whether testing patients for *APOL1* risk will affect clinical care processes, including appropriate kidney disease screening, or improve outcomes, such as BP control or incident CKD, or how patients will respond to testing. Effective communication of genetic risk to clinicians supported by clinical decision support in electronic health records (EHRs), as well as to patients, may be useful because it has been useful for hypertension control in general.^18,19^

Testing of *APOL1* status is being used in niche clinical settings, including for evaluation of living kidney donors.^20,21^ To our knowledge, there are no proven interventions at this time, but there appears to be consensus that the presence of high-risk alleles alone does not necessarily lead to kidney failure. Other factors, such as social determinants of health or control or lack of control of BP, may influence the clinical course of persons with high-risk alleles. To determine whether risk disclosure would affect BP and kidney testing, researchers collaborated with a genomics stakeholder board (consisting of patients, clinicians, advocates, and health system leaders) to conduct the Genetic Testing to Understand and Address Renal Disease Disparities (GUARDD) trial to study the effects of incorporating *APOL1* genotype information into primary care management of adults of African ancestry with hypertension.

## Methods

### Study Design and Participants

This randomized clinical trial (protocol in Supplement 1) enrolled patients from 15 academic, community, and safety-net practices in 2 health systems in New York City from November 1, 2014, through November 28, 2016; the final date of follow-up was January 16, 2018.^22^ Inclusion criteria were self-identified African ancestry, being 18 to 70 years of age, hypertension EHR diagnosis and/or taking antihypertensive medications and/or 2 systolic BP (SBP) readings greater than 140 mm Hg at least 6 months apart, community dwelling, English speaking, and receiving primary care at a participating site in the past year. Exclusion criteria were diabetes, CKD, pregnancy, moving away during the study period, and cognitive impairment. This study was approved by the Icahn School of Medicine for Family Health Institutional Review Board. All participants provided written informed consent. This study followed the Consolidated Standards of Reporting Trials (CONSORT) reporting guideline.^23^

### Recruitment of Participants

We used EHRs to identify patients potentially meeting inclusion criteria. Coordinators mailed these patients a recruitment letter, telephoned those who did not decline, screened them, and scheduled enrollment. We placed study flyers in waiting rooms, intercepted potentially eligible patients at clinics when they were hard to reach, and encouraged clinicians to refer patients.

### Randomization and Masking

We randomly assigned patients to immediate vs delayed intervention (waiting list control) in a 7:1 ratio so that the number of patients with 2 high-risk *APOL1* alleles would approximate the number of control patients to optimize study power. We stratified randomization by clinical site.

### Collection of Data

During baseline, 3-month, and 12-month study visits, trained coordinators administered a survey ascertaining demographic characteristics, knowledge, attitudes, and behaviors^22^; measured sitting BP with BpTru BPM-200 digital monitors validated against in-office and 24-hour ambulatory BPs; and averaged the second and third of 3 measurements.^24^ We identified urine protein to creatinine ratio and microalbumin to creatinine ratio testing in EHRs up to 12 months before (baseline) and for 12 months after (postintervention) randomization. For genetic testing at baseline for intervention patients and at 12 months for controls, coordinators collected venous blood samples (or saliva samples via Oragene kits if needed).

### *APOL1* Testing, Return of Results, and Clinical Decision Support

Our clinical decision support engine provided privacy-protected data flow and real-time communication between the EHR, genetic testing laboratory, and study team.^22^ A validated assay interrogated *APOL1* G1 and G2 alleles^7^ in a Clinical Laboratory Improvement Amendments, College of American Pathologists–accredited clinical laboratory. Coordinators trained and supervised by a senior genetic counselor returned results to ensure that all patients would receive standardized information in a timely manner. Patients received a result of high-risk *APOL1* genotypes if they were homozygous or compound heterozygous carriers of G1 and/or G2 alleles, and patients received a result of low-risk *APOL1* genotypes if they were heterozygous G1 or G2 or homozygous wild-type allele carriers. Coordinators returned results of low-risk *APOL1* genotypes over the telephone and results of high-risk *APOL1* genotypes in person, using “teach back” (coordinators asked patients to repeat their explanation in the patients’ own words, and if not correct, coordinators would repeat or reframe the explanation until the patients were correct) to ensure comprehension.^25^ All patients received a scripted message indicating that it is important to control BP for many reasons, including kidney health; were asked if they wanted to speak to or meet with the study’s genetic counselor; and received a low literacy educational booklet with their results.

Return of results triggered an EHR best practice alert with results that fired once for every primary care professional (PCP) who opened an encounter in the 1-year follow-up period.^26^ Waiting list control patients received the results after their 12-month follow-up visit. The alert included the result, a message that BP control can help forestall kidney failure, the 3 most recent BP readings, and links to information for clinicians and patients with 1-click access and printing. The entire process was informed by formative interviews; developed in partnership with the genomic stakeholder board’s patients, advocates, and clinicians; tailored by and for patients of African ancestry and for low-literate populations; and extensively piloted and revised.^27^

### Outcomes

We compared patients with high-risk *APOL1* genotypes with patients with low-risk *APOL1* genotypes for the 2 primary end points: 3-month SBP and 12-month urine albumin screening for kidney disease. Secondary outcomes included differences in SBP and urine testing in an enriched intervention group (patients with high-risk *APOL1* genotypes) vs controls and psychobehavioral patient factors between groups and over time. We did not focus on kidney screening via serum creatinine testing because it is part of routine panels ordered for reasons often unrelated to CKD screening or assessment.

### Statistical Analysis

Statistical analysis was performed from May 1, 2018, to July 31, 2020. We used mean (SD) values to describe continuous variables and proportions to describe categorical variables; the *t* test or analysis of variance was used to compare continuous variables, and the χ^2^ test or the Fisher exact test was used to compare categorical variables by groups. To test significance of changes within groups over time, we used paired *t* tests for continuous variables and McNemar tests for categorical variables. We used linear mixed models to test the difference in SBP change over time between patients with high-risk *APOL1* genotypes and those with low-risk *APOL1* genotypes by entering the interaction term of time and *APOL1* status and adjusting for confounders, and we used generalized estimating equation methods to test the change in controlled SBP status and kidney function testing over time between patients with high-risk *APOL1* genotypes and those with low-risk *APOL1* genotypes and similarly between patients with high-risk *APOL1* genotypes and controls. Analyses were conducted in SAS, version 9.4 (SAS Institute Inc), and *P* values were from 2-sided tests, with significance set at *P* < .05.

## Results

From November 1, 2014, to November 30, 2016, we approached 5481 patients identified in EHR queries; 2050 (37%) were enrolled, 2783 (51%) were ineligible, 234 (4%) declined, and 412 (8%) were undecided when enrollment finished (Figure). Of 2052 enrolled, 2050 were randomized. Participants had a mean (SD) age of 53 (10) years; 1360 of 2050 were women (66%; a similar proportion as in the initial EHR list), 1014 of 2050 (50%) had very low income, 769 of 2050 (38%) had low health literacy, and 530 of 2050 (26%) had very good or excellent self-rated health (Table 1). We retained 92% (1886 of 2050) at 3 months and 77% (1587 of 2050) at 12 months, with no differences in follow-up rates by study group.^28^ There were no differences in sociodemographic characteristics between intervention and control patients except that control patients had higher educational attainment (more than some college: 162 of 255 [64%] control patients vs 1010 of 1795 [56%] intervention patients). However, patients with high-risk *APOL1* genotypes had significantly higher mean (SD) SBP at baseline (137 [21] mm Hg) than those with low-risk *APOL1* genotypes (134 [19] mm Hg; *P* = .003) and controls (133 [19] mm Hg; *P* = .001) (Table 2).

**Figure.**
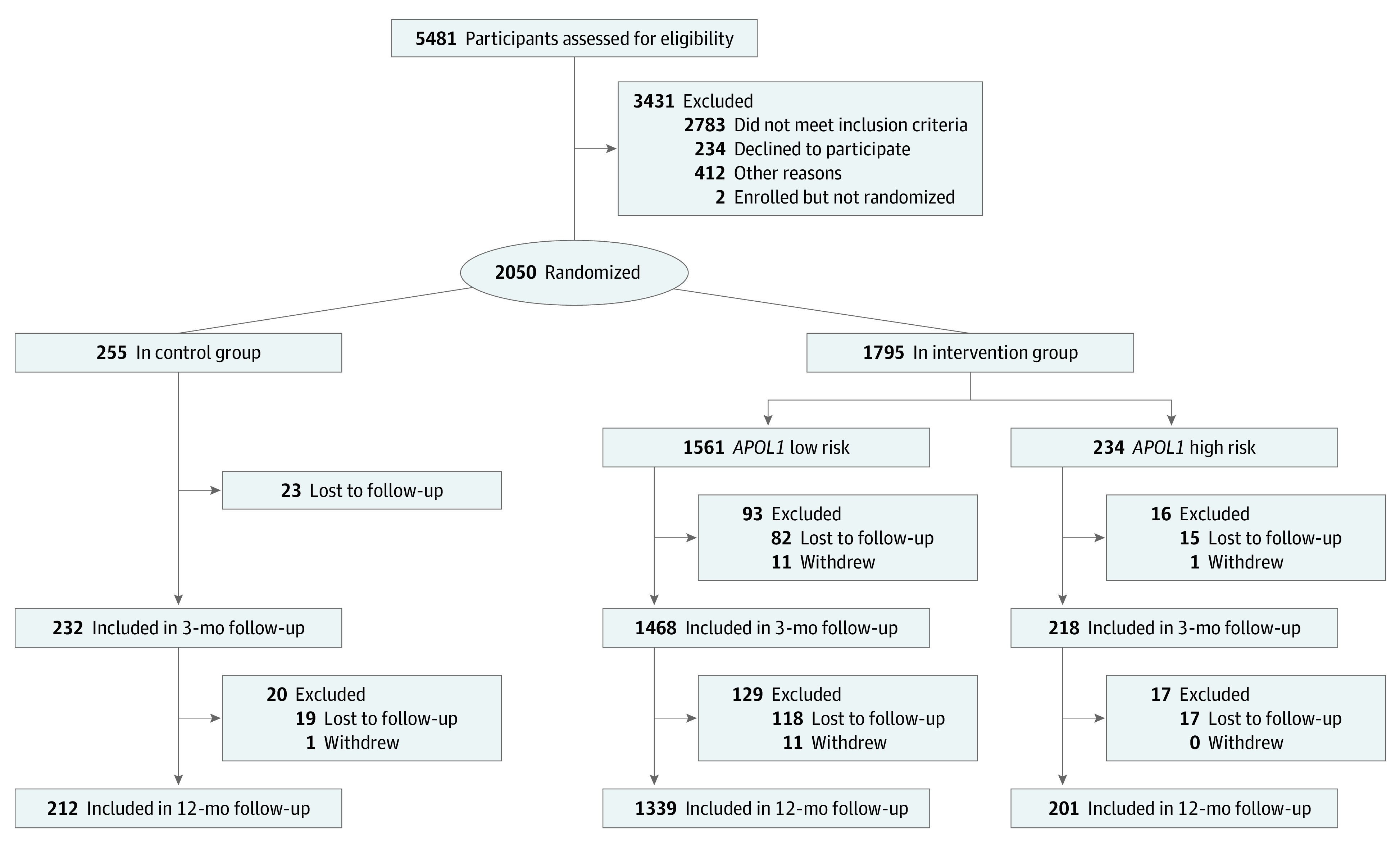
Enrollment and Randomization of Study Participants

**Table 1.** Characteristics of Patients by Randomization and *APOL1* Status Groups

Characteristic	Patients, No. (%)	
	Delayed intervention, control (n = 255 [12%])	Intervention (n = 1795 [88%])
Age, mean (SD), y	53 (10)	53 (10)
Male	83 (33)	607 (34)
Female	172 (67)	1188 (66)
Household income <$30 000/y	126 (49)	888 (50)
Education more than some college	162 (64)	1010 (56)
Married or living with partner	72 (28)	541 (30)
Insurance		
Private	118 (46)	747 (42)
Medicaid	109 (43)	821 (46)
Medicare	14 (5)	126 (7)
Other	8 (3)	51 (3)
None	6 (2)	50 (3)
Charlson Comorbidity Index		
0	141 (55)	925 (52)
1	61 (24)	481 (27)
≥2	53 (21)	391 (22)
Baseline mean (SD), mm Hg		
Systolic blood pressure	133.8 (19.4)	134.2 (19.6)
Diastolic blood pressure	85.4 (12.2)	86.4 (12.3)
BMI, mean (SD)	33.9 (7.7)	32.4 (7.7)
High health literacy	169 (66)	1112 (62)
Excellent or very good self-reported health	64 (25)	466 (26)

Abbreviation: BMI, body mass index (calculated as weight in kilograms divided by height in meters squared).

**Table 2.** Main Outcome Comparisons Between Randomization and *APOL1* Status Groups

Outcome	Control (n = 255 [12%])	Intervention		Unadjusted *P* value		Adjusted *P* value^a^
		Low-risk genotypes (n = 1561 [76%])	High-risk genotypes (n = 234 [11%])	High-risk *APOL1* genotypes vs low-risk *APOL1* genotypes	High-risk *APOL1* genotypes vs control	
SBP, mean (SD), mm Hg						
At baseline	133 (19)	134 (19)	137 (21)	.003	.03	.02
At 3 mo	131 (19)	131 (18)	131 (19)	.83	.92	
Difference in SBP from baseline to 3 mo, mean (SD)	−3 (18)	−3 (18)	−6 (18)	.004	.01	
*P* value for change over time within group	.02	<.001	<.001	NA	NA	
Kidney function urine test, No. (%)						
Before randomization	33 (13)	278 (18)	39 (17)	.67	.25	.24
After randomization	50 (20)	377 (24)	68 (29)	.10	.01	
*P* value for change over time within group	.01	.01	<.001	NA	NA	

Abbreviations: NA, not applicable; SBP, systolic blood pressure.

^a^ For blood pressure, the *P* values for the interaction term of time × *APOL1* status were adjusted for age, sex, baseline SBP, body mass index, comorbidity, income, educational level, and marital status among intervention patients from the linear mixed model. For kidney function urine test, *P* values for the interaction term of time × *APOL1* status were adjusted for age, sex, body mass index, insurance, type of clinician (attending physician or fellow, resident, or nurse practitioner), and institution (academic vs community).

### Change in SBP at 3 Months

Although all groups had some decrease in SBP, it was greatest in patients with high-risk *APOL1* genotypes (mean [SD], 6 [18] mm Hg) vs those with low-risk *APOL1* genotypes (mean [SD], 3 [18] mm Hg; *P* = .004) or controls (mean [SD], 3 [18] mm Hg; *P* = .01) (Table 2). The percentage change in SBP was significantly different between patients with high-risk *APOL1* genotypes (3.6%), those with low-risk *APOL1* genotypes (1.0%; *P* = .003), and controls (1.3%; *P* = .04). After adjustment for age, sex, body mass index, comorbidity, income, educational level, and marital status, the decrease in SBP remained statistically significantly different between patients with high-risk *APOL1* genotypes and those with low-risk *APOL1* genotypes (*P* = .02). The mean (SD) diastolic BP at 3 months was 83.6 (12.3) mm Hg in patients with high-risk *APOL1* genotypes, 83.8 (10.9) mm Hg in those with low-risk *APOL1* genotypes, and 83.7 (10.9) mm Hg in controls. There was no significant difference in SBP at 12 months or in the change in SBP from baseline to 12 months between groups.

### Urine Testing for Kidney Function at 12 Months

A similar proportion of patients in all 3 groups had urine protein excretion tests at baseline. At 12 months after the intervention, all 3 groups showed a significant increase in the rate of urine protein testing over time. The most significant increase was seen in patients with high-risk *APOL1* genotypes (12% increase: from 39 of 234 [17%] to 68 of 234 [29%]) compared with those with low-risk *APOL1* genotypes (6% increase: from 278 of 1561 [18%] to 377 of 1561 [24%]) and controls (7% increase: from 33 of 255 [13%] to 50 of 255 [20%]), a difference significant between patients with high-risk *APOL1* genotypes and controls (*P* = .01), although, over time across groups, this difference did not remain statistically significant (Table 2). Thus, the prespecified urine testing outcome was negative; however, a significant difference was seen for the patients with high-risk *APOL1* genotypes vs controls on nonprespecified analysis.

### Psychobehavioral Outcomes of Patients

A total of 1468 of 1561 patients with low-risk *APOL1* genotypes and 218 of 234 patients with high-risk *APOL1* genotypes completed the surveys. Significantly more patients with high-risk *APOL1* genotypes than patients with low-risk *APOL1* genotypes reported making positive lifestyle changes (a subjective measure that included better dietary and exercise habits) after receiving *APOL1* results (129 of 218 [59%] vs 547 of 1468 [37%]; *P* < .001), changing how they take their BP medications (53 of 218 [24%] vs 146 of 1468 [10%]; *P* < .001) and taking BP medications more often (21 of 218 [10%] vs 68 of 1468 [5%]; *P* = .005) (Table 3). Nearly all patients stated that they had enough information to decide about getting the test, the information was easy to understand, and they would get tested again. Few had negative reactions, but more patients with high-risk *APOL1* genotypes were upset about their results (17 of 218 [8%] vs 15 of 1468 [1%]; *P* < .001) and worried they would develop kidney problems (59 of 218 [27%] vs 249 of 1468 [17%]; *P* < .001). We offered all patients the opportunity to speak to or meet with a genetic counselor at no cost; however, none chose to do so.

**Table 3.** Comparison by *APOL1* Status on Patient Psychosocial Behaviors 3 Months After Randomization

Characteristic	Intervention *APOL1*, No. (%)^a^		*P* value
	Low-risk genotypes (n = 1468)	High-risk genotypes (n = 218)	
Had enough information on getting the test	1425 (97)	208 (95)	.28
Information for test was easy to understand	1391 (95)	203 (94)	.42
Would get tested again	1420 (97)	211 (97)	.83
Made positive lifestyle changes^b^	547 (37)	129 (59)	<.001
Changed how you take BP medication	146 (10)	53 (24)	<.001
Take BP medication more often	68 (5)	21 (10)	.005

Abbreviation: BP, blood pressure.

^a^ A total of 1468 of 1561 patients with *APOL1* low-risk genotypes and 218 of 234 patients with *APOL1* high-risk genotypes completed the surveys.

^b^ Lifestyle changes were subjective and included having better dietary and exercise habits.

## Discussion

Chronic kidney disease and its complications are at epidemic levels,^29,30,31^ and BP control is important for reducing the incidence and progression of CKD.^32^ In partnership with the genomics stakeholder board, we recruited and tested adults with self-reported African ancestry and hypertension for *APOL1* alleles that increase the risk of CKD and end-stage kidney disease. We returned genetic test results to them through trained laypersons and to their PCPs via EHR alerts at 15 academic, community, and safety-net practices. Disclosure of *APOL1* genetic risk results led to a greater decrease in SBP among those with high-risk *APOL1* genotypes vs those with low-risk *APOL1* genotypes and among those with high-risk *APOL1* genotypes vs controls, as well as a significantly greater increase in urine kidney disease screening in all groups, especially among patients with high-risk *APOL1* genotypes. Patients had favorable reactions to testing and return of results, and patients with high-risk *APOL1* genotypes reported positive changes in lifestyle and BP medication use compared with patients with low-risk *APOL1* genotypes.

This trial tested the effects of disclosing *APOL1* genetic results to patients of African ancestry with hypertension and their PCPs. Baseline SBP was higher in patients with high-risk *APOL1* genotypes (as previously reported^33^), but the SBP absolute and percentage improvement was higher in this group vs those with low-risk *APOL1* genotypes or controls. To our knowledge, few studies disclosing genetic risk of chronic diseases have led to improved outcomes.^34,35^ Our findings may be due to the use of EHR alerts, which have previously shown efficacy,^18,19^ along with disclosure of genetic results to patients. The magnitude of SBP improvement was small, but we did not provide specific BP target recommendations or BP-lowering strategies to clinicians or patients; future programs may show more benefit with more intense BP-lowering efforts. The SBP improvement may also be the result of the change in behaviors among the patients with high-risk *APOL1* genotypes. Health behavior changes were small and, although statistically significant, may have been clinically not meaningful. The increase in urine screening across all groups may be because physicians were aware of being part of a clinical trial and thus may have changed their test-ordering patterns in response. However, this outcome was modified by an awareness of patients’ genetic risk, with a higher increase in kidney disease screening among patients with high-risk *APOL1* genotypes vs those with low-risk *APOL1* genotypes.

Translational genomics and clinical informatics may play increasing roles in primary care–based chronic disease prevention and control efforts, but the effect of returning results needs to be tested in rigorous randomized clinical trials. Primary care professionals are central to helping patients understand and address risk and interactions between clinical, genetic, and environmental factors, and they will likely be crucial in efforts to minimize the risk and maximize the benefit of genetic testing in diverse populations and settings.^36^ Thus, return of genetic risk results to PCPs with clear care plans, targeted education, and recommended clinical workflows would increase knowledge about and acceptability of genomic medicine in primary care. This study contributes to the evidence base that knowledge of genetic risk in primary care may affect processes and outcomes of care.

To date, the optimal way to return genetic results to patients with common diseases in primary care settings is not clear. Genetic counselors have been on the front line of delivering genetic testing results to patients and clinicians regarding mendelian disorders, but PCPs are on the front line of health care for most patients. Genetic counselors are a limited resource^37^ and may not be critical in returning results for chronic disease risk. Our stakeholders and formative research^27,38^ guided us to have patients tested and receive results in their primary care practices via laypersons who were mainly of African ancestry and trained by genetic counselors to return results and by local stakeholders to be sensitive to the culture and challenges facing patients. We did not rely on PCPs to return test results because they did not order the test and did not feel prepared to return results,^39^ but we provided them with decision support to discuss results with their patients. This approach was well received by the patients; none chose to meet with a genetic counselor, although we offered the opportunity to all, and they viewed disclosure of results very favorably. Clinician extenders (nurse practitioners and physician assistants) may be useful for other practices to consider to permanently fill this role for busy clinicians or as a bridge while clinicians learn to incorporate information from a relatively new field into their workflow.

### Limitations

The study has some limitations. We excluded patients with CKD, and it is important to study the effects of genetic testing and disclosure of results on patients with abnormal kidney function. Our intervention had a modest effect size, possibly owing to increase in medication use and/or lifestyle change, which may have substantial benefits at the population level. However, interventions with more robust components and repeated reminders may demonstrate a more significant effect. Our primary outcome was within the intervention group, although we did see effects when comparing patients with high-risk *APOL1* genotypes vs controls. In addition, we did not have comprehensive data on lifestyle and dietary intake or medication refill data. In this pilot trial, the period of outcome assessment was short (1 year). However, this trial has informed a national multicenter trial called GUARDD-US, which is ongoing. We used change in SBP as a primary outcome because of prior strong associations with cardiovascular disease.^40^ However, diastolic BP is an important risk factor and will be explored in future work. We did not have information on treatment fidelity collected as part of the study protocol, and we did not have significant information on the type and dosage of medication and could only address the changes in broad groups. The upcoming GUARDD-US trial will address these issues in more detail. Confounding (including lifestyle factors, kidney function, and severity and treatment of hypertension) could affect these results. Finally, although we conducted this trial in academic, clinical, and primary care settings, we did so in 1 urban area, and it will be important to validate the findings in other settings.

## Conclusions

Return of *APOL1* genetic testing results combined with EHR-based clinical decision support and disclosure of results to patients using laypersons improved SBP control and increased guideline-appropriate kidney function testing. These results may support an approach of broad implementation of genetic medicine in primary care. This broad implementation will benefit racial and ethnic minority groups that have been traditionally underrepresented in both clinical trials and genetic studies.^41^ Because it is imperative not to overlook the importance of social determinants of health in affecting chronic disease, it will also be important to understand and address the intersection of social and biological determinants in patient health.

## Notes

Supplement 1.
Trial Protocol

Supplement 2.
Data Sharing Statement
