# Supplementary material for: Effects of Testing and Disclosing Ancestry-Specific Genetic Risk for Kidney Failure on Patients and Health Care Professionals: A Randomized Clinical Trial
Source: JAMA Netw Open. 2022 Mar 4;5(3):e221048. doi: 10.1001/jamanetworkopen.2022.1048 (PMC8897752; doi:10.1001/jamanetworkopen.2022.1048)
Supplement: Supplement 1. — Trial Protocol [file jamanetwopen-e221048-s001.pdf]

# Genetic testing to Understand and Address Renal Disease Disparities (GUARDD)

**Principal Investigator:** Carol R. Horowitz, MD, MPH

**ClinicalTrials.gov Identifier:** NCT02234063

**Protocol version:** March 16, 2016

**TABLE OF CONTENTS**


| | | |
|---|---|---|
| 1. | BACKGROUND AND SIGNIFICANCE | 3 |
| 1.1 | Overview | 3 |
| 1.2 | Study Aims | 5 |
| 2. | ENDPOINTS | 7 |
| 2.1 | Primary Endpoints | 7 |
| 2.2 | Secondary Endpoints | 7 |
| 3. | STUDY DESIGN | 8 |
| 3.1 | Study Arms & Design | 8 |
| 3.2 | Randomization | 8 |
| 3.3 | Blinding | 9 |
| 3.4 | Sample Size | 9 |
| 3.5 | CCARP Genomics Subcommittee- Stakeholder Engagement | 9 |
| 4. | STUDY POPULATION | 9 |
| 4.1 | Inclusion Criteria | 9 |
| 4.2 | Exclusion Criteria | 10 |
| 5. | RECRUITMENT AND ENROLLMENT PROCEDURES | 10 |
| 5.1 | Participating Sites | 10 |
| 5.2 | Provider Recruitment, Consent and Survey | 10 |
| 5.3 | Participant Identification and Recruitment Strategies | 10 |
| 5.4 | Screening Procedures | 11 |
| 5.5 | Participant Consent Process | 11 |
| 5.6 | Participant Discontinuation/Withdrawal from the Study | 12 |
| 5.7 | Lost to Follow-Up | 12 |
| 5.8 | Risks | 12 |
| 5.9 | Benefits | 13 |
| 5.10 | Costs to the Participants | 13 |
| 5.11 | Compensation to Participants | 13 |
| 6. | STUDY PROCEDURES | 13 |
| 6.1 | Provider Surveys | 13 |
| 6.2 | Baseline Study Visit | 13 |

| | | |
|---|---|---|
| 6.3 | Follow-Up Assessments | 14 |
| 6.4 | Specimen Collection | 15 |
| 6.5 | Specimen Transfer and Genetic Testing Procedures | 15 |
| 6.6 | Return of Genetic Results | 15 |
| 6.7 | Electronic Health Record Data Extraction | 16 |
| 6.8 | Study Retention | 18 |
| 7. | SAFETY ASSESSMENT AND MONITORING | 18 |
| 7.1 | Distress from Return of Results | 18 |
| 7.2 | Elevated Blood Pressure Readings | 18 |
| 8. | STATISTICAL ANALYSIS PLAN AND SAMPLE SIZE | 18 |
| 8.1 | Sample Size Determination | 18 |
| 8.2 | General Statistical Methods | 19 |
| 8.3 | Population for Analyses | 19 |
| 8.4 | Analysis of the Secondary Endpoints | 19 |
| 8.5 | Handling of Missing Data | 19 |
| 9. | DATA MANAGEMENT | 19 |
| 9.1 | Data Entry and Record Keeping | 19 |
| 9.2 | Database Management and Quality Control of Data | 20 |
| 10. | ETHICAL AND HUMAN SUBJECTS CONSIDERATIONS | 20 |
| 10.1 | Institutional Review Board | 20 |
| 10.2 | Large Scale Data Sharing | 20 |
| 11. | PROTOCOL DEVIATIONS | 21 |
| REFERENCES | | 22 |


# 1. BACKGROUND AND SIGNIFICANCE

## 1.1 Overview

Approaches and experiences of ongoing early adopter programs for incorporating genomic information in clinical care have to date been largely limited to examples conducted at a small group of academic institutions in few highly-specialized areas, including pharmacogenetics (1, 2), tumor-based screening (3, 4), family history-based decision support (5, 6), and diagnostic whole exome/genome sequencing (4, 7). However, studies that develop robust systems to consent, screen, return results, and that evaluate processes and outcomes of incorporating genomic risk information in clinical care for common chronic diseases are missing and urgently

needed. We propose that hypertension-attributable chronic kidney disease has emerged as a highly-relevant opportunity for a 'prototype' genomic medicine demonstration project that addresses common chronic illnesses managed in primary care settings. Hypertension-attributable chronic kidney disease (CKD) is characterized by

- high prevalence affecting millions of Americans (8),
- high burden of morbidity and mortality related mainly to increased cardiovascular disease risk and kidney failure or end stage renal disease (8),
- progression to kidney failure that can be modified by appropriate pharmacological interventions (9-11),
- a disproportionate burden for African Ancestry and major health disparity (12) (13-16) (17) (18),
- a substantial and testable population selective genomic risk that explains most of the excess burden of hypertension-attributable CKD risk in African Ancestry populations (19) (20) (21) (22).

**Synthesis of evidence and formulation of study rationale**

**Synthesis: Chronic Kidney Disease (CKD), Hypertension and Blood Pressure Control.** CKD is a common, complex disease affecting 26 million Americans adults (8). CKD is most commonly attributable to diabetes (40% of CKD cases) and hypertension (28% of cases). African Ancestry populations with hypertension (HTN) have 2- to 3-fold higher risk of developing CKD, and a 5-fold increased risk to progress to end stage renal disease (ESRD) when compared with whites. HTN is an established risk factor for progression of CKD and for increased cardiovascular risk with CKD. Thus targeting blood pressure control as a modifiable risk factor may both reduce CVD in people with CKD and reduce progression of CKD to end stage disease (9-11).

**Synthesis: Poor adherence with renal care practice guidelines puts participants at risk for kidney failure**
Importantly, major goals of practice guidelines for renal care in hypertensive participants remain unmet in clinical practice today: among Medicare participants with hypertension without diabetes, only 1 in 25 receives recommended simple lab tests (creatinine and urine albumin) to evaluate CKD, and less than half of all participants with moderate to advanced stages of CKD in the Kidney Early Evaluation Program (KEEP) are aware that they are affected (23). Among younger participants of African Ancestry CKD awareness is particularly low (23) and progression to kidney failure is typically accelerated resulting in excessive rates of ESRD (13) (18).

Improved CKD awareness and access to primary care or nephrology referral for individuals with or at risk of CKD are considered critical to improve CKD-related outcomes (24, 25). Factors associated with progression of CKD and with increased cardiovascular risk are overlapping to a large extent, including hypertension. There is strong evidence that blockade of the renin-angiotensin system is a blood pressure lowering strategy which is more effective in reducing risk of kidney and cardiovascular disease in the presence of albuminuria, a marker of CKD (26). Thus, in order to improved renal care and reduce risk for kidney failure in this population at excess risk, we urgently need new strategies:

- to improve comprehension of CKD risk and CKD awareness among participants with CKD or at risk for CKD and among their providers, and
- to increase adherence with practice guidelines targeting those risk factors that are modifiable may both reduce cardiovascular disease in people with CKD and reduce progression of CKD to end stage kidney disease.

**Synthesis: *APOL1* G1 and G2 risk alleles and non-diabetic kidney diseases.**
A locus containing the myosin heavy chain 9 (MYH9) gene for non-diabetic kidney disease in African ancestry individuals was initially identified by admixture mapping (27, 28). Recently, three non-synonymous coding

variants in the neighboring *APOL1* gene defined two allele, termed G1 and G2 with stronger effect on non-diabetic kidney disease than MYH9 variants (19). The authors suggested that G1 and G2 alleles are exceedingly rare in non-African ancestry genomes, but in African ancestry genomes, 22.5% and 14.6% of chromosomes carry the mutually-exclusive G1 and G2 risk alleles because they were selected for by providing protection against Trypanosomiasis (sleeping sickness) in West Africa (19).

*APOL1*-associated kidney disease risk is best explained using a recessive model, and approximately 13% of African Americans are estimated homozygous for G1 / G2 risk alleles, suggesting that more than 3 million AA are at markedly increased risk for non-diabetic CKD (20). In our IPM Biobank, 15% of more than 5,000 AA participants were found to carry [2] risk alleles, and the odds for hypertensive CKD in this cohort were 2.7. We recently analyzed the effect of APOL risk alleles on severity of hypertension-attributed kidney disease in the African American Study of Kidney Disease and Hypertension (AASK) cohort (Table 1). *APOL1* risk alleles are highly significantly associated with CKD attributed to essential hypertension in non-diabetic AASK participants, and the odds for advanced kidney disease (significant proteinuria or serum creatinine >3 mg/dl) were >4-fold in carriers of [2] risk alleles compared to [0,1] risk alleles (22). Heterozygous G1 or G2 risk allele status does not appear to increase kidney disease risk.

**Table 1**. Logistic regression model of the effect of APOL1 risk alleles on clinical phenotype AASK cases and controls

Note: creatinine >2 or >3 mg/dL approximate CKD Stage 3 or higher; ESKD indicates end stage kidney disease, i.e. CKD stage 5; urine PCR (protein creatinine ratio) > 0.22 g/g or > 0.60 g/g correlate approximately with albumin creatinine ratio (ACR) of > 30 mg/g and > 300 mg/g.

In summary, [2] *APOL1* G1 / G2 homozygous risk allele status

- explains practically all of the substantial excess genetic risk for non-diabetic CKD in African ancestry populations,
- is present in 1 out of 7 African American participants genotyped at Mount Sinai,
- is strongly and consistently associated with hypertension-attributable CKD (odds >2.7)
- is strongly and consistently associated with progressive and proteinuric states of hypertension-attributable CKD (odds >4.0) or with hypertensive ESRD (odds 7.3).

Thus, published evidence and our own Mount Sinai results strongly support our hypothesis that carriers of [2] *APOL1* risk alleles have an increased genomic risk for hypertension-attributable CKD and its progression to kidney failure.

## 1.2 Study Aims

GUARDD is multifaceted and has elements that involve qualitative research for a formative study to better information development of the randomized trial. This protocol is focused on the randomized controlled trial.

**AIM II. Develop systems and evidence-based advice messages to enable point of care Clinical Decision Support (CDS) for primary care providers advising renal care practice guidelines with or without genomic *APOL1* risk information**

**Rationale.** One of the highly anticipated quality improvement advantages offered by EHRs and 'meaningful use' is the potential for point of care Clinical Decision Support (CDS). CDS provides clinicians or participants with knowledge presented at appropriate times to improve healthcare. In this context, CDS also has the potential to increase the awareness of and adherence to, standard of care processes. Mount Sinai's IPM conducts several early adopter projects testing utility and adoption of pharmacogenomic CDS for clinicians in real-time at the point of care. We will develop new functionality for our existing CLIPMERGE Risk Assessment Engine database (CRAE database) to deliver CDS for renal care practice guidelines based on conventional kidney disease risk assessment with or without *APOL1* genomic kidney disease risk information. Importantly, we will for the first time develop interfaces that will allow CRAE to disseminate standardized CDS to independent Epic EHR implementations across different primary care practice settings at IFH and MSMC.

**Sub-Aim 2.1. Modification of CLIPMERGE Risk Assessment Engine (CRAE) technology for multiple EHR (IFH and MSMC) and renal care CDS capabilities.** CRAE houses phenotypic and gene variant data necessary for the evaluating enrolled participants' data relevant to the guidelines to be implemented for this study. The CRAE database will be populated only for enrolled participants. The CRAE database itself houses very strictly de-identified data (only). A separate function named the Broker handles all necessary translation between identified data (as needed for participant enrollment, for transactions flowing from and to Epic, and for receipt of genomic results from the CLIA lab) and de-identified data.
The **CLIPMERGE-EPIC Integration.** The CLIPMERGE database will include longitudinal clinical data extracted from Mount Sinai's and IFH's Epic EHR systems, for all consented participants enrolled in the research study; including CLIA-grade *APOL1* genotype and G1 G2 risk allele data from those that have been genotyped. Our CLIPMERGE Risk Assessment Engine (CRAE) includes this database and a rules engine that relates genome-based advice messages (renal care advice messages incorporating *APOL1* genomic risk information with conventional risk data) or conventional risk based advice messages to standard of care clinical decision support messages. During the first six months of year 1, the CLIPMERGE and Epic team at Mount Sinai will work with the Epic team at IFH to build HL7 interfaces customized between CRAE and the specific Epic version installed for IFH sites.
**Reference and educational material.** Upon presentation of a BPA, providers will have the opportunity to directly access reference content that further describes the evidence base for the CDS through a clickable link in the Epic SmartSet.

**Sub-Aim 2.2. Development and usability testing of a library of evidence-based renal care advice messages customized for assessment of risk with and without *APOL1* G1 G2 risk allele information, and for adherence to practice guidelines for renal care in non-diabetic African ancestry participants with hypertension**

**Rationale.** CKD awareness among participants and providers, appropriate use of tests for biochemical markers of CKD (creatinine and urinary albumin excretion) to screen for presence of CKD in those at risk, and appropriate use of pharmacological and life style interventions in those at risk for CKD progression are considered critical to improve CKD-related outcomes (ESRD, CVD, and mortality (24, 25). As summarized in the paragraph "**Synthesis: Poor adherence with renal care practice guidelines puts participants at risk for kidney failure"** at the beginning of the APPROACH section, major goals of practice guidelines for renal care in hypertensive participants remain unmet in clinical practice today. Blacks have higher prevalence of hypertension (41% vs. 28%), younger age of onset, and poorer control of hypertension than Whites (17). Blacks also have, a 2-3x the risk for developing CKD (12), and the adjusted prevalence rate for ESRD is 4.1-fold higher in AA when compared with Whites (14-16). The differences in CKD are most pronounced among those with hypertension. Thus, it is imperative to uncover new strategies to screen and engage AAs with hypertension into programs to improve BP control and participant outcomes.

The Kidney Disease Improvement Global Outcomes (KDIGO) evidence-based practice guidelines advise the appropriate use of tests for biochemical markers of CKD (creatinine and urinary albumin excretion) to screen for presence of CKD in those at risk, and appropriate use of pharmacological and lifestyle interventions in those at risk for CKD progression are considered critical to improve CKD-related outcomes (ESRD, CVD, and mortality (24, 25). Practice guidelines, including the Joint National Commission 7 (JNC7) guidelines, recommend specific medications (ACE inhibitors and ARBs) as preferred first line agents and more intensive blood pressure goal in participants with hypertensive CKD (9-11).

Several reports demonstrate that *APOL1* risk alleles are highly significantly associated with CKD attributed to essential hypertension in non-diabetic AASK participants, and the genetic association was most robust in individuals with progressive renal functional decline (22) (see our AASK Cohort data in Table 1). Because of the overwhelming strengths of the evidence, we propose to establish *APOL1* genomic risk status in non-diabetic AA participants and to incorporate the *APOL1* G1/G2 risk allele status in a recessive model together with conventional risk factors in CKD and CKD progression advice messages.

**<u>Aim III</u>. Conduct a randomized trial assigning eligible participants to immediate genetic testing or delayed genetic testing arms in a seven (immediate testing) -to- one (delayed testing) ratio.**

Sub-Aim 3.1: To examine whether increase in practice guideline-appropriate renal laboratory test ordering (renal care endpoint) will be achieved in *APOL1*-positive group vs *APOL1*-negative group.

Sub-Aim 3.2. To examine whether systolic blood pressure will decline more in the *APOL1*-positive group compared with *APOL1*-negative group.

## 2. ENDPOINTS

### 2.1 Primary Endpoints

The study has two primary endpoints, comparing patients who are *APOL1* positive (high risk) and *APOL1* negative at three months after enrollment. One primary aim is a renal care endpoint, the correct utilization, by clinicians, of urine albumin tests. The other primary aim is reduction of systolic blood pressure.

### 2.2 Secondary Endpoints

Secondary endpoints include differences between APOL1 positive participants in the intervention and participants in the control group, impact on primary outcomes at 12 months, psycho-behavioral differences of participants between groups and over time, clinician knowledge, attitudes and beliefs at baseline and 12 months, and differences in outcomes between those tested and not tested immediately.

## 3. STUDY DESIGN

### 3.1 Study Arms & Design

This is a prospective, multicenter, unblinded, randomized clinical trial (RCT) (**Figure 1**). The study was designed to randomize 2050 participants to immediate *APOL1* gene testing and return of results (ROR) (intervention) or delayed *APOL1* gene testing and ROR (control) in a 7:1 ratio. Outcome measures will be compared among three arms of the GUARDD study, the *APOL1*-positive and *APOL1*-negative intervention (immediate *APOL1* genetic testing and return of results) groups, and the control (delayed *APOL1* testing and return of results) group.

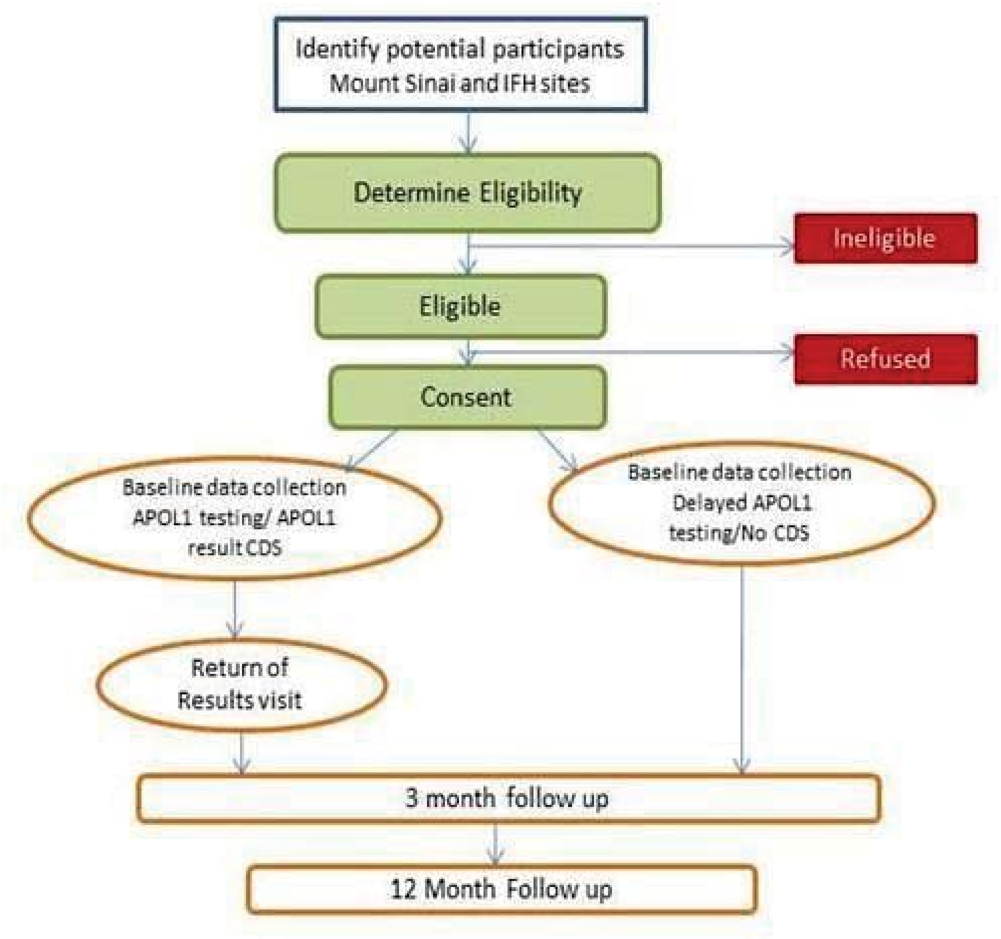


**Figure 1.** GUARDD study flowchart.

## 3.2 Randomization

Eligible participants will be randomized in a 7:1 allocation to Intervention (i.e., immediate *APOL1* gene testing and ROR to participant and provider) and Control arms (delayed *APOL1* gene testing and ROR to participant and provider) to optimize the proportion of participants with immediate *APOL1* genetic testing compared with delayed genetic testing at completion of study. Randomization will be stratified by clinical site with a random block size within site.

## 3.3 Blinding

GUARDD randomization assignments will not be blinded to any participants, providers or study personnel. To minimize bias in the measurement of the primary outcome, randomization assignments will only be revealed after baseline survey responses and blood pressure readings have been collected. Digital blood pressure devices (such as BpTru portable blood pressure machine (29)) will be used to measure blood pressure, and blood pressure will be measured as the mean of the second and third blood pressure readings for each participant at each study visit.

## 3.4 Sample Size

The total number of people expected to participate is 2,050. Of the 1800 intervention participants, approximately 250 will test positive. We will thus have approximately 250 high risk, 250 control, and 1550 normal risk participants. The sample size for the study was calculated assuming a 10% improvement in practice guideline-appropriate renal function test ordering in the *APOL1*-positive group (40% estimated) vs. *APOL1*-negative group (30% estimated) to yield 87% power to detect the difference of interest using a two-sided chi-square test. Specifically, this includes measurement of serum creatinine and urine microalbumin. The blood pressure sub-aim, 5mmHg improvement in systolic blood pressure at 3-month follow up in *APOL1*-positive compared with *APOL1*-negative group, can be detected with 95% power.

## 3.5 CCARP Genomics Subcommittee- Stakeholder Engagement

The CCARP Genomics Subcommittee members comprised of clinicians, researchers and community leaders will be in every stage of the research, including: choosing the study approach, tailoring and shaping the patient education materials, developing and implementing recruitment and retention strategies, deciding what to evaluate with survey questions, and disseminating our findings to the community.

# 4. STUDY POPULATION

## 4.1 Inclusion Criteria

- Self-reported African American/Black or having African Ancestry
- English speaking
- Age 18-70 years
- Have diagnosis of hypertension
  - Diagnosis of hypertension is defined by either:
    - ICD9 diagnosis codes (present in encounter diagnosis or in problem list) and/or
    - Taking anti-hypertensive medications or
    - 2 systolic blood pressure readings >140 mm Hg or 2 diastolic readings >90 at least six months apart.
- Received primary care from one of the participating clinical sites ≥1 within the past 2 years.
- Do not have diabetes by self report, or defined by:
    - ICD9 diagnosis codes (present in encounter diagnosis or in problem list) or
    - HbA1c ≥ 6.5 at least one time in the last year
- Do not have CKD by self report or defined by either:
    - 1) ICD9 codes OR
    - GFR < 60 ml/min

## 4.2 Exclusion Criteria

- Have diabetes measured or by self report
- Have CKD measured or by self report
- Pregnancy at time of enrollment by self report

- Too cognitively impaired to provide informed consent and/or complete the study protocol measured by mini mental status exam
- Institutionalized or too ill to participate (i.e. terminally ill, incarcerated, in psychiatric or nursing home facility) by self report
- Plan to move out of the area within 12 months of enrollment by self report
- Not a patient under the care of a provider for their hypertension at a participating site by self report
- Previously participated in the *APOL1* qualitative pilot study or have previously undergone *APOL1* testing

# 5. RECRUITMENT AND ENROLLMENT PROCEDURES

## 5.1 Participating Sites

Recruitment will be from primary care clinics within Mount and Institute for Family Health.

## 5.2 Provider Recruitment, Consent and Survey

We will present the study to providers at participating clinics and ask them to complete a short form consent explaining the purpose and the voluntary nature of their participation in the study. They will also receive the GUARDD APOL1 Provider Baseline Survey to be completed upon enrollment where we will ask questions about their current knowledge of genomics and personalized medicine and clinical decision support. At this time, we will also ask providers to give us permission to contact their patients who may be eligible to participate in the randomized trial and to have a recruitment letter sent to their patients signed by them or their practice. Providers who prefer to have a recruitment letter sent from them will provide a signature on a copy of the letter. As new providers join practices, we will also obtain their permission to contact their patients and ask them to complete the survey.

We will ask providers at participating sites to complete the GUARDD APOL1 12 Month Follow-Up Survey approximately 12 months after study enrollment. This questionnaire will contain similar questions asked at baseline, in addition to reactions to any clinical decision aids or exposure to APOL1 genetic testing over the course of the study. A list of participating providers will be kept by the study team to track completion of questionnaires and training attendance at the different study sites.

## 5.3 Participant Identification and Recruitment Strategies

<u>Participant Identification</u>

Electronic Health Record (EHR) Data Runs for Eligible Participants

Potential participants will be primarily identified through data runs using the inclusion criteria mentioned above. Lists with PHI identifying potential participants are emailed to the Program Manager via a secure, password protected Excel file. Participant's, date of birth, address and phone numbers, primary care provider name and clinic site extracted from the EHR are imported into the Redcap study database.

Referral

Participants may self-refer from any advertisement materials (posters, flyers). They may also be referred by a family member or friend, or by a provider who has agreed to allow their participants to participate in the study.

If a participant is referred, the Study Coordinator should check the REDcap database to ensure the participant is not already in the database and proceed accordingly.

Participant Recruitment

There are three main scenarios for recruitment:

- **Via recruitment letter:** Study Coordinators will be assigned participants (from the list of potentially eligible participants obtained through an Epic data run) and mail study recruitment letters to them. Letters are mailed in a bright blue envelope and postmarked with the GUARDD and clinic site logo to assist participants with recall and recognition. Participants can call us upon receiving the letter and they will then be screened for study eligibility. Study Coordinators will wait approximately 2 weeks after this mailing for a participant to return the letter refusing to participate, to call them, or to meet them at an upcoming clinic visit.
- **During a phone call:** If a participant does not return the refusal letter or contact us within 2 weeks of mailing, the Study Coordinator assigned to him/her will contact the participant by telephone. Using the recruitment phone script they will remind them about the letter that was mailed to them, introduce or reintroduce the study, screen them for eligibility, and answer any questions about the study.
- **During a clinic visit:** The Program Manager will receive a weekly list of which participants/potentially eligible participants have a scheduled clinic appointment and will notify the Study Coordinator assigned to that clinic site so s/he will plan to meet the participant at that time. The clinical Study Coordinator will discuss the study with the participant, screen them for eligibility, and answer any questions about the study.

Study Coordinators will then schedule all eligible and interested participants for a baseline visit. A baseline visit may occur at the time of recruitment (if approached at a clinic visit) or at a later date.

### 5.4 Screening Procedures

Study Coordinators will use a recruitment script during recruitment phone calls or clinic intercepts to inform the potential participant about the study and screen participants for eligibility using the study inclusion/exclusion criteria. If the participant is eligible and interested, a baseline visit will be scheduled that same day or for a future date.

### 5.5 Participant Consent Process

If a participant is interested and eligible, Study Coordinators will review the consent document at the start of the baseline study visit. Prospective research participants will have the opportunity to ask questions before providing written consent. Study Coordinators will provide the participant with a copy of the consent document. If the individual chooses not to sign the consent form, the Study Coordinator will inform him/her they are unable to participate in the study. The participant will also be provided the option to be contacted for future research.

### 5.6 Participant Discontinuation/Withdrawal from the Study

Participants may stop participating or withdraw from the study at any point in time. All information and data collected from the participant up to that point can be used in the study. Withdrawal of consent to participate in

the research study can be verbal or in writing. Study Coordinators should attempt to obtain a reason for withdrawal from the participant and record it in the study database.

Participants may withdraw from the study at any time by writing to the PI or by verbally informing the study coordinator or Program Manager. Any data collected up until withdrawal may be analyzed for study purposes but no new information will be collected.

### 5.7 Lost to Follow-Up

Losses to follow-up may be minimized and retention maximized through various mechanisms, including offering study visits during evening and weekend hours, collecting information for and contacting family members when participants cannot be reached, approaching research participants at clinic appointments, and completing surveys over-the-phone (although research participant should still attend study visits for blood pressure measurement). Certified letters may also be sent to those not reached by phone. Study Coordinators can confirm the best contact information for the participant at each study visit. Study Coordinators may also obtain permission to contact participants via text message or email and may send additional correspondence during the study. Participants may be assigned to a specific Study Coordinator at the site in order to maintain continuity and build rapport.

### 5.8 Risks

This research presents minimal risks to participants. Possible risks are described below.

Blood Pressure
Participants may feel some arm pressure when the blood pressure cuff is briefly inflated.

Blood Draw
The risks of a blood draw include pain, bruising, and the slight possibility of infection at the location of needle insertion. Some participants may feel dizzy or may faint during or after a blood draw.

Saliva Collection
Some people may feel discomfort because they cannot eat, drink, smoke or chew gum for 30 minutes before giving a saliva sample.

Psychological Distress Learning of Test Results
Results of the genetic test may show that a participant is at an increased risk of kidney disease. This knowledge may cause anxiety or psychological distress. Study staff will be trained to recognize anxiety and psychological stress and talk through this discomfort with the participant. All participants will have the option to speak with a genetic counselor if they choose.

### 5.9 Benefits

Participants may not receive any benefit from taking part in this research.  Others may not benefit either. However, participants and their providers will obtain clinically-relevant genomic risk information to guide evaluation and treatment of hypertension and renal functioning, thus providing some indirect benefit to participant health.

### 5.10 Costs to the Participants

The costs of study-related genomic testing are covered by the study and will not be billed to participants. Taking part in this research study may lead to minor added costs including, for example, transportation to attend study visits. Participants (and/or their health care payer) will still be billed for the costs of their regular medical care that are not part of this study.

### 5.11 Compensation to Participants

Study participants will receive $40 in gift cards (to a variety of local retailers) at each of the Baseline, 3 Month and 12 Month follow-up visits. If a participant withdraws from the study before all visits are completed, they will be paid for any completed visits. If a participant is able to complete a survey over the phone for a follow up visit, but is unable to come in-person for a blood pressure measurement within the visit window, they will only receive a $20 gift card, which may be mailed to them at the address they provide.

## 6. STUDY PROCEDURES

### 6.1 Provider Surveys

Prior to first enrollment at a site, at one of their existing meetings, providers will be asked to complete a consent form to contact their potentially eligible participants and to complete the Baseline Provider Survey to assess demographics, knowledge, beliefs and practices around *APOL1* testing specifically and genetic testing more generally. This survey is anonymous. A GUARDD clinical champion will present the study to the providers of each site

### 6.2 Baseline Study Visit

Consent

The Study Coordinator will follow appropriate consenting protocol to consent interested and eligible participants.

Survey

After participants sign the informed consent, the Study Coordinators will confirm participant contact information and administer the GUARDD Baseline Survey using the REDCap database. Study Coordinators also have the option of administering surveys on paper and entering the responses in the RedCap database at a later date.

Biological Measures

Study Coordinators will obtain blood pressure using study specific protocols (outlined in the MOP) for blood pressure measurement using the BPTru portable blood pressure machine (29). If after several attempts, the study coordinator is unable to obtain an arm blood pressure measurement because the participant’s arm is too large for the arm cuff or another reason, blood pressure will be measured using the Omron HEM 670IT wrist monitor and make a note in the participant’s Redcap record. Study Coordinators will record the second and third blood pressure measurement in which will calculate the average blood pressure reading.

Study Coordinators will also measure participant height using the Charder HM200P portable stadiometer (at Baseline Visit only) and weight using the Detecto DR550C portable high capacity platform scale and input this information into the RedCap database which will calculate BMI.

Study Coordinators will record the average blood pressure and participant height and weight on the Personal Health Screening Form along with estimated dates for study follow up for the participant's personal records.

If the participant has a blood pressure reading greater than 190/110 during the study visit, study staff must complete an Elevated Blood Pressure Note for the participant, record the reading on REDCap, inform participant that they have very high blood pressure and strongly advise that they get urgent and appropriate evaluation and care from a healthcare provider. Study staff must also inform study PIs.

Specimen Collection

Participants will be randomized via the REDCap randomization tool using a stratified randomization scheme by clinical site in a 7:1 ratio of immediate or delayed *APOL1* genetic testing. The Study Coordinator informs the participant of their randomization outcome. A blood (preferred) or saliva sample is collected from participants randomized to immediate testing by the Study Coordinator. Study Coordinators will obtain a genetic sample for control participants at their 12 month visit. Participants are informed that their result will be ready in 2-4 weeks.

The baseline visit will take approximately 1.5 hours to complete. Participants will receive $40 in gift cards when they have completed their baseline visit.

## 6.3 Follow-Up Assessments

All participants will be advised that they will be contacted to complete a follow-up study visit at 3 months and 12 months after enrollment (duration of the observation period). During follow-up visits, Study Coordinators will follow study protocol (outlined in the MOP) to conduct surveys, measure blood pressure, and obtain participant weight in order to calculate body mass index. They will enter this data directly into REDCap using tablets/laptops. The follow-up visits will take approximately 45 minutes to complete. Participants will receive a $40 gift card for each follow-up visit completed.

Follow-up visits will be completed between 14 days prior and one month after the projected follow-up date (projected 3 month study visit due date = 3 months from date of baseline visit completion; projected 12 month study visit due date = 12 months from date of baseline visit completion). Whenever possible, participants will meet with the same Study Coordinator for follow-up visits to maintain continuity.

If the participant has a blood pressure reading greater than 190/110 during any follow up visit, study staff must complete an Elevated Blood Pressure Note, inform the participant that they have very high blood pressure and strongly advise that they get urgent and appropriate evaluation and care from a healthcare provider. Study staff must also inform study PIs.

## 6.4 Specimen Collection

Blood Specimen: At the baseline visit, Study Coordinators will consent participants if not previously consented, administer the baseline survey, take 3 blood pressure readings, measure participant height and weight and, if

trained in phlebotomy, collect 1 purple tops EDTA tube (approximately 3-5 mL) of venous blood from participants willing to provide a blood sample. If the Study Coordinator is not trained in phlebotomy, the participant will have their blood drawn by a trained phlebotomist. Participants will then be randomized to the Control or Intervention arm. Study Coordinators will label the tube with the participant ID, date of birth, sex and the date sample was collected, and follow the MOP for the collection, storage, and delivery of the sample to the laboratory.

Saliva Specimen: In the event that it is not possible to obtain a blood sample or participants prefer saliva collection, Study Coordinators will use an Oragene OG-500 kit to collect a saliva sample. They will label the sample and follow the saliva collection protocol for the collection, storage, and delivery of the sample to the laboratory as outlined in the MOP**.**

## 6.5 Specimen Transfer and Genetic Testing Procedures

Study Coordinators should store and directly transport specimens to the Mount Sinai Genetics Testing Laboratory, a Clinical Laboratory Improvement Amendments (CLIA)-certified laboratory, according to procedures outlined in the MOP. Specimens will be accepted by the lab twice a week and processed on a weekly basis.

The *APOL1* G1/G2 genotype testing incorporates Polymerase Chain Reaction (PCR) and multiplex Allele Specific Primer Extension (ASPE) with Tm Bioscience's proprietary Universal Tag sorting system on the Luminex® 100 xMAP™ platform. Three SNP genotype in exon 6 are tested (RS73885319, RS60910145, RS71785313) to determine G1 and G1 allele status. To validate this genotyping method, at least three intra-assay and inter-assay runs involving 50 positive controls and 8 negative controls were performed. The positive control DNAs include samples which are heterozygous or homozygous for G1 and G2 genotypes. Negative controls are WT DNA for G1 and G2 alleles. Assays were 100% concordant reproducibly between the Luminex genotyping method and Sanger sequencing for each of the control samples.

## 6.6 Return of Genetic Results

Result reporting will occur approximately 1-4 weeks after the samples are obtained (see Fig. 1 Study flow chart). The laboratory will notify the study Program Manager of when results are ready to be returned. The Program Manager will in turn alert Study Coordinators. Return of results will be done by their assigned Study Coordinators. Study Coordinators will be trained by Randi Zinberg, Director of Mount Sinai Genetic Counseling Program, to return risk assessment results and recommendations. They will use a Return of Results Script for this visit. *APOL1* negative participants will have their tests results returned by phone (participants in the formative study said they did not want to come back in person for a negative results) and those that are *APOL1* positive will be scheduled for an in person return of results visit. If an in person return of results visit is not able to be scheduled for an *APOL1* positive participant, we will offer the participant the opportunity to receive their results over the phone. During return of results, the Study Coordinator will disclose the genetic test result, provide simple, clear information and use "speak back" or "teach back" technique to maximize participants' comprehension of their result. In addition to verbal ROR, participants will also receive lay explanations of their test results in writing and the educational booklet about *APOL1*, blood pressure and kidney

disease. *APOL1* positive participants will review the informational booklet at the time of their return of results, *APOL1* negative participants will receive their written test results and informational booklet in the mail.

If we are unable to return an *APOL1* positive test result to the participant for at least 3 months we will notify their primary care provider so that the result can be noted in their electronic health record and the participant can be notified of their results when they get in contact with their provider, even if that is after the study has ended.

All participants will be given the option to speak to a genetic counselor after their return of results in person or by telephone, at no charge. If a participant chooses to speak with a genetic counselor, the Study Coordinator will contact the genetic counselor on behalf of the participant, and the genetic counselor will then follow-up directly with the participants.

Once a result is returned to the participant, the Study Coordinator completes the corresponding fields in Redcap that prompts CLIPMERGE to fire a Best Practice Alert (BPA) in the participant's electronic health record the next time a primary care provider opens it. The BPA will pop up once per unique provider. A provider can choose to open the BPA and has the option of clicking on and viewing and printing information for the participant and information for him/herself. In addition to the BPA, the lab will also place a copy of the *APOL1* genetic test results in the participant's electronic medical record. Electronic Health Record Data Extraction

### 6.7 Electronic Health Record Data Extraction

Electronic health data relevant to the study endpoints will be pulled for the period of 12 months prior to randomization and 12 months after randomization for all enrolled participants.

**Figure 2**. *APOL1* positive BPA Alert

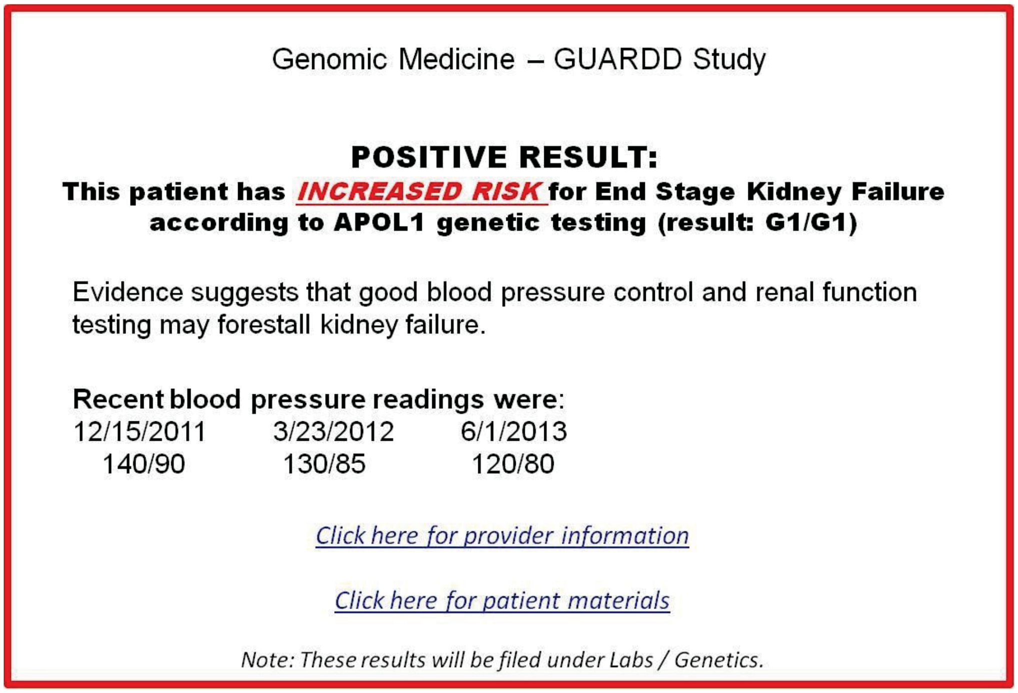

## 6.8 Study Retention

Carefully trained, dedicated Study Coordinators that are from the same demographic groups and neighborhoods as participants will recruit participants, and will facilitate retention using relationship building, continuity with their assigned participants, sending personalized birthday and holiday cards, sending a 6 month check in postcard, placing a 9 month check in phone call, and collecting multiple contacts and modes of contact (e.g., phone, mail, text, email, intercepting at upcoming clinical appointments) from study participants. They will also "intercept" participants at clinical visits should they have clinical visits during the follow-up windows. If participants are unable to come to the practice for the entire visit, they can be surveyed by phone and come for blood pressure check, and if they cannot come at all, they can be surveyed by phone.

# 7. SAFETY ASSESSMENT AND MONITORING

The GUARDD Study is an observational-type study that does not include a drug or device intervention. For this reason, no adverse events will be collected or recorded in the study database. Adverse events suspected to be related to study interventions should be reported to the Mount Sinai and Institute for Family Health IRBs according to their local policies.

## 7.1 Distress from Return of Results

Distress from return of results will be monitored at participant visits. If the participant seems overly distressed by the outcomes of the genetic test results, the Research Coordinator will offer the participant the opportunity to speak to the genetic counselor. If the genetic counselor is not immediately available, the Research Coordinator will help coordinate a phone call or meeting with the genetic counselor based on participant's preference. The study coordinator will also inform the Project Manager and PI, document the event in the notes field of the participant's study database record, and follow-up with participant.

## 7.2 Elevated Blood Pressure Readings

If systolic blood pressure exceeds 190 mm Hg or diastolic exceeds 110 mm Hg during any study visit, the participant will be strongly advised to seek urgent and appropriate evaluation and care from a healthcare provider. The Research Coordinator may facilitate this by assisting the participant in contacting their primary care provider, urgent care, or clinic staff on site including the Principal Investigator (if study visit is taking place in a clinical setting) and by completing the Elevated Blood Pressure Note that participants may share with their provider.

# 8. STATISTICAL ANALYSIS PLAN AND SAMPLE SIZE

## 8.1 Sample Size Determination

The study will randomize 2050 patients to either immediate or delayed genetic testing in a ratio of 7:1 respectively. We anticipate that of the 1800 patients who will be tested immediately approximately 250 will test positive. For this aim, we hypothesize that APOL1 positive patients will achieve 40% correct utilization of serum creatinine and/or urine albumin tests for "standard renal care" in patients with hypertension, compared to

30% for APOL1 negative patients. With an estimated 250 APOL1 positive and 1550 APOL1 negative we will have 87% power to detect the difference of interest using a two sided chi-square test.

Power calculations for this sub-aim are based on reduction of systolic blood pressure at three months after enrollment. For patients who test APOL1 positive we anticipate a 5 mmHg reduction in systolic blood pressure, compared to no change in patients who test APOL1 negative. Assuming that the standard deviation for differences in blood pressure between baseline and 3 month in both arms is 20 mmHg, a total of 250 patients testing positive and 1550 testing negative provides approximately 95% power to detect a difference of 5 mmHg in SBP between these two groups. If the standard deviation is larger than assumed, say 25 mmHg, power will still exceed 80%. Power is based on a 0.05 level two-tailed t-test of the difference in SBP at 3 months.

### 8.2 General Statistical Methods

We will use mean and standard deviation to describe continuous variables, proportions for categorical variables; t-tests or analysis of variance (ANOVA) to compare continuous variables, and chi-square or Fisher's exact test to compare categorical variables by groups. To test significance of changes within groups over time, we will use paired t-tests for continuous and McNemar's tests for categorical variables. We will use linear mixed models to test the difference in SBP change over time between *APOL1* positives and negatives by entering the interaction term of time and *APOL1* status and adjusting for confounders, and generalized estimating equation methodology to test the change in controlled SBP status and renal function testing overtime between *APOL1* positives and negatives, and similarly between *APOL1* positives and controls.

### 8.3 Population for Analyses

Patients will be from academic, community and safety-net practices in New York City. Inclusion criteria are: self-identified AA; age 18–70 years; hypertension EHR diagnosis and/or taking antihypertensive medications, and/or 2 SBP readings >140mmHg at least six months apart; community-dwelling; English speaking; and receiving primary care at participating site in the past year. Exclusion criteria are: diabetes; CKD; pregnancy; moving away during the study period; and cognitive impairment.

### 8.4 Analysis of the Secondary Endpoints

Secondary outcomes include differences in SBP and urine testing in an enriched intervention group (*APOL1* positives) vs. controls, and psycho-behavioral patient factors between groups and over time.

### 8.5 Handling of Missing Data

Missing data will be analyzed as intention to treat.

## 9. DATA MANAGEMENT

### 9.1 Data Entry and Record Keeping

Data will be entered and stored in a REDCap database to track and monitor participants. The database was adapted from the data dictionary to include MRNs and participant IDs, inclusion criteria, baseline, 3- and 12-month participant contact logs and surveys, calendar and reminder functions, and ability for recruiters,

managers and investigators to track workflow and perform queries to assess the status of participants (i.e., who is outstanding for a 3-month ROR1 visit).

### 9.2 Database Management and Quality Control of Data

A REDCap database will be developed to track and monitor participants. This includes MRNs and participant IDs, inclusion criteria, baseline, 3- and 12-month participant contact logs and surveys, calendar and reminder functions, and ability for recruiters, managers and investigators to track workflow and perform queries to assess the status of participants (i.e., who is outstanding for a 3-month visit). Data will be entered into REDCap using tablets/laptops. Study staff will be trained on how to enter data and will receive a unique user identification and password to access data entry forms for their site. Access codes should not be shared and are non-transferable. Study Coordinators will always have paper survey copies as backup should Redcap be down or they experience technical difficulties.

Genetic test results are uploaded directly into the REDCap database through a CLIPEMERGE interface and verified by the Program Manager. The database includes password protection and internal quality checks, such as automatic range limits and regular checks to identify data that appear inconsistent, incomplete, or inaccurate. The Program Manager and study biostatistician will review the data on a regular basis as part of quality control. The check will review the data for errors, outliers, missing fields, inconsistencies, etc.

## 10. ETHICAL AND HUMAN SUBJECTS CONSIDERATIONS

### 10.1 Institutional Review Board

This study will be initiated only after all required documentation has been reviewed and approved by the Mount Sinai and Institute for Family Health Institutional Review Boards (IRBs).

### 10.2 Large Scale Data Sharing

The sharing of our dataset will follow the requirements set forth by the NIH policy for data sharing and guidelines for NIH Data Set Preparation. The de-identified and anonymized phenotype and genotype data will may be made available in NIH’s database of Genotypes and Phenotypes (dbGaP) repository for sharing to the larger scientific community.

Databases, like dbGAP, were created to meet the needs of the medical genetics community by storing medical information from many studies conducted at many different places. Researchers can then study the combined information to learn even more about health and many different diseases. Some databases are publicly accessible and some are restricted. Anyone on the Internet can access the information shared in publicly accessible databases. However, only researchers who apply to restricted databases and are approved can access databases, like dbGAP. The current study will limit sharing of data to only those databases, which are restricted and require approval to access, like dbGAP that maintain Certificates of Confidentiality.

## 11. PROTOCOL DEVIATIONS

A protocol deviation is defined as an event where the Investigator or site personnel did not conduct the study according to the protocol. Protocol deviations will be reported to the GUARDD Program Manager, Principal Investigator and Mount Sinai and Institutes for Family Health IRB according to their policies.

## REFERENCES


1. Pulley, J.M., Denny, J.C., Peterson, J.F., Bernard, G.R., Vnencak-Jones, C.L., Ramirez, A.H., Delaney, J.T., Bowton, E., Brothers, K., Johnson, K., et al. 2012. Operational Implementation of Prospective Genotyping for Personalized Medicine: The Design of the Vanderbilt PREDICT Project. *Clinical pharmacology and therapeutics* 92:87-95.
2. Crews, K.R., Cross, S.J., McCormick, J.N., Baker, D.K., Molinelli, A.R., Mullins, R., Relling, M.V., and Hoffman, J.M. 2011. Development and implementation of a pharmacist-managed clinical pharmacogenetics service. *American journal of health-system pharmacy : AJHP : official journal of the American Society of Health-System Pharmacists* 68:143-150.
3. Gudgeon, J.M., Williams, J.L., Burt, R.W., Samowitz, W.S., Snow, G.L., and Williams, M.S. 2011. Lynch syndrome screening implementation: business analysis by a healthcare system. *The American journal of managed care* 17:e288-300.
4. Worthey, E.A., Mayer, A.N., Syverson, G.D., Helbling, D., Bonacci, B.B., Decker, B., Serpe, J.M., Dasu, T., Tschannen, M.R., Veith, R.L., et al. 2011. Making a definitive diagnosis: successful clinical application of whole exome sequencing in a child with intractable inflammatory bowel disease. *Genetics in medicine : official journal of the American College of Medical Genetics* 13:255-262.
5. Khoury, M.J., Feero, W.G., and Valdez, R. 2010. Family history and personal genomics as tools for improving health in an era of evidence- based medicine. *American journal of preventive medicine* 39:184-188.
6. Orlando, L.A., Hauser, E.R., Christianson, C., Powell, K.P., Buchanan, A.H., Chesnut, B., Agbaje, A.B., Henrich, V.C., and Ginsburg, G. 2011. Protocol for implementation of family health history collection and decision support into primary care using a computerized family health history system. *BMC health services research* 11:264.
7. Bainbridge, M.N., Wiszniewski, W., Murdock, D.R., Friedman, J., Gonzaga-Jauregui, C., Newsham, I., Reid, J.G., Fink, J.K., Morgan, M.B., Gingras, M.C., et al. 2011. Whole-genome sequencing for optimized participant management. *Science translational medicine* 3:87re83.
8. Coresh, J., Selvin, E., Stevens, L.A., Manzi, J., Kusek, J.W., Eggers, P., Van Lente, F., and Levey, A.S. 2007. Prevalence of chronic kidney disease in the United States. *JAMA* 298:2038-2047.
9. Chobanian, A.V., Bakris, G.L., Black, H.R., Cushman, W.C., Green, L.A., Izzo, J.L., Jr., Jones, D.W., Materson, B.J., Oparil, S., Wright, J.T., Jr., et al. 2003. Seventh report of the Joint National Committee on Prevention, Detection, Evaluation, and Treatment of High Blood Pressure. *Hypertension* 42:1206-1252.
10. Kidney Disease Outcomes Quality Initiative (K/DOQI). 2004. K/DOQI clinical practice guidelines on hypertension and antihypertensive agents in chronic kidney disease. *Am J Kidney Dis.* 43:S1-290.
11. Padwal, R.S., Hemmelgarn, B.R., Khan, N.A., Grover, S., McKay, D.W., Wilson, T., Penner, B., Burgess, E., McAlister, F.A., Bolli, P., et al. 2009. The 2009 Canadian Hypertension Education Program recommendations for the management of hypertension: Part 1--blood pressure measurement, diagnosis and assessment of risk. *The Canadian journal of cardiology* 25:279-286.
12. Muntner, P., Newsome, B., Kramer, H., Peralta, C.A., Kim, Y., Jacobs, D.R., Jr., Kiefe, C.I., and Lewis, C.E. 2012. Racial differences in the incidence of chronic kidney disease. *Clinical journal of the American Society of Nephrology : CJASN* 7:101-107.

13. Centers for Disease Control and Prevention (CDC). 2007. Racial differences in trends of end-stage renal disease, by primary diagnosis--United States, 1994-2004. *MMWR Morb Mortal Wkly Rep.* 56:253-256.
14. Hsu, C.Y., Lin, F., Vittinghoff, E., and Shlipak, M.G. 2003. Racial differences in the progression from chronic renal insufficiency to end-stage renal disease in the United States. *J Am Soc Nephrol* 14:2902-2907.
15. Tomson, C.R., Foley, R.N., Li, Q., Gilbertson, D.T., Xue, J.L., and Collins, A.J. 2008. Race and end-stage renal disease in the United States Medicare population: the disparity persists. *Nephrology (Carlton)* 13:651-656.
16. Freedman, B.I., Langefeld, C.D., Rich, S.S., Valis, C.J., Sale, M.M., Williams, A.H., Brown, W.M., Beck, S.R., Hicks, P.J., and Bowden, D.W. 2004. A genome scan for ESRD in black families enriched for nondiabetic nephropathy. *J Am Soc Nephrol* 15:2719-2727.
17. Hertz, R.P., Unger, A.N., Cornell, J.A., and Saunders, E. 2005. Racial disparities in hypertension prevalence, awareness, and management. *Archives of internal medicine* 165:2098-2104.
18. Tarver-Carr, M.E., Powe, N.R., Eberhardt, M.S., LaVeist, T.A., Kington, R.S., Coresh, J., and Brancati, F.L. 2002. Excess risk of chronic kidney disease among African-American versus white subjects in the United States: a population-based study of potential explanatory factors. *J Am Soc Nephrol* 13:2363-2370.
19. Genovese, G., Friedman, D.J., Ross, M.D., Lecordier, L., Uzureau, P., Freedman, B.I., Bowden, D.W., Langefeld, C.D., Oleksyk, T.K., Uscinski Knob, A.L., et al. 2010. Association of trypanolytic ApoL1 variants with kidney disease in African Americans. *Science* 329:841-845.
20. Friedman, D.J., Kozlitina, J., Genovese, G., Jog, P., and Pollak, M.R. 2011. Population-based risk assessment of APOL1 on renal disease. *Journal of the American Society of Nephrology : JASN* 22:2098-2105.
21. Birchenall-Roberts, M.C., Ruscetti, F.W., Kasper, J., Lee, H.D., Friedman, R., Geiser, A., Sporn, M.B., Roberts, A.B., and Kim, S.J. 1990. Transcriptional regulation of the transforming growth factor beta 1 promoter by v-src gene products is mediated through the AP- 1 complex. *Mol.Cell Biol.* 10:4978-4983.
22. Lipkowitz, M.S., Freedman, B.I., Langefeld, C.D., Comeau, M.E., Bowden, D.W., Kao, W.H.L., Astor, B.C., Bottinger, E.P., Iyengar, S.K., Klotman, P.E., et al. 2012. Apolipoprotein L1 gene variants associate with hypertension-attributed nephropathy and the rate of kidney function decline in African Americans. *Kidney international* In Press.
23. Agrawal, V., Jaar, B.G., Frisby, X.Y., Chen, S.C., Qiu, Y., Li, S., Whaley-Connell, A.T., McCullough, P.A., and Bomback, A.S. 2012. Access to health care among adults evaluated for CKD: findings from the Kidney Early Evaluation Program (KEEP). *American journal of kidney diseases: the official journal of the National Kidney Foundation* 59:S5-15.
24. Saab, G., Whaley-Connell, A.T., McCullough, P.A., and Bakris, G.L. 2008. CKD awareness in the United States: the Kidney Early Evaluation Program (KEEP). *American journal of kidney diseases: the official journal of the National Kidney Foundation* 52:382-383.
25. Plantinga, L.C., Boulware, L.E., Coresh, J., Stevens, L.A., Miller, E.R., 3rd, Saran, R., Messer, K.L., Levey, A.S., and Powe, N.R. 2008. Participant awareness of chronic kidney disease: trends and predictors. *Archives of internal medicine* 168:2268-2275.

26. Appel, L.J., Wright, J.T., Jr., Greene, T., Agodoa, L.Y., Astor, B.C., Bakris, G.L., Cleveland, W.H., Charleston, J., Contreras, G., Faulkner, M.L., et al. 2010. Intensive blood-pressure control in hypertensive chronic kidney disease. *The New England journal of medicine* 363:918-929.
27. Kao, W.H., Klag, M.J., Meoni, L.A., Reich, D., Berthier-Schaad, Y., Li, M., Coresh, J., Patterson, N., Tandon, A., Powe, N.R., et al. 2008. MYH9 is associated with nondiabetic end-stage renal disease in African Americans. *Nat Genet* 40:1185-1192.
28. Kopp, J.B., Smith, M.W., Nelson, G.W., Johnson, R.C., Freedman, B.I., Bowden, D.W., Oleksyk, T., McKenzie, L.M., Kajiyama, H., Ahuja, T.S., et al. 2008. MYH9 is a major-effect risk gene for focal segmental glomerulosclerosis. *Nat Genet* 40:1175-1184.
29. Wright, J. M., Mattu, G. S., Perry, T. L., Jr, Gelferc, M. E., Strange, K. D., Zorn, A., & Chen, Y. 2001. Validation of a new algorithm for the BPM-100 electronic oscillometric office blood pressure monitor. *Blood pressure monitoring*, *6*(3), 161–165. https://doi.org/10.1097/00126097-200106000-00008

# Baseline Survey

Record ID ______________________

Research history collected:
Recruitment: [baseline_arm_1][research_history]
Survey Baseline: [baseline_arm_1][research_history_baseline]
Survey 3 mo: [followup_3_mo_arm_1][research_history_3m]
Call 9 mo: [followup_12_mo_arm_1][research_history_9_mo]
Survey 12 mo: [followup_12_mo_arm_1][research_history12m]

If 'Yes/No' in any of the categories above, skip 1st question.

1. Have you ever taken part in another research study other than this one?
   - ◯ Yes
   - ◯ No

Q10 Overall, would you say your health in general is excellent, very good, good, fair, poor or very poor

- ◯ Excellent
- ◯ Very good
- ◯ Good
- ◯ Fair
- ◯ Poor
- ◯ Very poor
- ◯ DON'T KNOW
- ◯ REFUSED

## Now we'd like to ask you about having high blood pressure.

Q 20 Do you have a family member who has had high blood pressure? (parent, grandparent, brother, sister, child)

- ◯ Yes
- ◯ No
- ◯ DON'T KNOW
- ◯ REFUSED

## IPQ- HTN

| | Not at all | A little bit | Somewhat | Very much | Extremely | DON'T KNOW | REFUSED |
|---|---|---|---|---|---|---|---|
| Q30 How much does having high blood pressure affect your life? | ◯ | ◯ | ◯ | ◯ | ◯ | ◯ | ◯ |
| Q40 Do you feel you have control over your high blood pressure? | ◯ | ◯ | ◯ | ◯ | ◯ | ◯ | ◯ |
| Q50 How much do you think your treatment can help your high blood pressure? | ◯ | ◯ | ◯ | ◯ | ◯ | ◯ | ◯ |

| | | | | | | | |
|---|---|---|---|---|---|---|---|
| Q60 How concerned are you about your high blood pressure? | ◯ | ◯ | ◯ | ◯ | ◯ | ◯ | ◯ |
| Q70 How much do you think that people inherit high blood pressure through their genes? | ◯ | ◯ | ◯ | ◯ | ◯ | ◯ | ◯ |

Now we would like to ask you some questions about medications you have been given or that you take.

Q75 Are you currently taking medications for your blood pressure or has a provider told you that you need to OR SHOULD take them?

◯ Yes, I am taking blood pressure medications
◯ No, I have been told I need to take them but I am not currently taking them.
◯ No, I have not been told to take blood pressure medications.
◯ 888 DON'T KNOW
◯ 999 REFUSED

**Outcomes Expectations- HTN**

**Please let me know if you agree or disagree with the following If participant not taking blood pressure medications because they have not been prescribed to him/her: Please answer the following questions thinking about how you would feel if you were told you needed to take blood pressure medications):**

| | Strongly Agree | Agree | Neither Agree Nor Disagree | Disagree | Strongly Disagree | DON'T KNOW | REFUSED |
|---|---|---|---|---|---|---|---|
| Q80 I (would)worry about the long-term effects of my blood pressure medications | ◯ | ◯ | ◯ | ◯ | ◯ | ◯ | ◯ |
| Q90 I (would)expect medication to control my high blood pressure | ◯ | ◯ | ◯ | ◯ | ◯ | ◯ | ◯ |
| Q100 I (would)will not have a stroke or heart attack if I take blood pressure medication | ◯ | ◯ | ◯ | ◯ | ◯ | ◯ | ◯ |
| Q110 I (would)will not have kidney problems or kidney failure if I take blood pressure medication | ◯ | ◯ | ◯ | ◯ | ◯ | ◯ | ◯ |
| Q120 I (would) expect to take blood pressure medication for the rest of my life | ◯ | ◯ | ◯ | ◯ | ◯ | ◯ | ◯ |
| Q130 I (would) take my blood pressure medication only when I have symptoms | ◯ | ◯ | ◯ | ◯ | ◯ | ◯ | ◯ |

| | | | | | | | |
|---|---|---|---|---|---|---|---|
| Q140 The medication (would) helps me reduce stress | ◯ | ◯ | ◯ | ◯ | ◯ | ◯ | ◯ |
| Q150 The blood pressure medication will (would) help me live longer | ◯ | ◯ | ◯ | ◯ | ◯ | ◯ | ◯ |
| Q160 I will (would) feel better if I take blood pressure medication | ◯ | ◯ | ◯ | ◯ | ◯ | ◯ | ◯ |
| Q170 I will (would) not need medication anymore when my blood pressure is normal | ◯ | ◯ | ◯ | ◯ | ◯ | ◯ | ◯ |

**Adapted from Beliefs About Medicines (bmq_18)**

**And, how much do you agree or disagree with the following statements:**

| | Strongly Agree | Agree | Neither Agree Nor Disagree | Disagree | Strongly Disagree | DON'T KNOW | REFUSED |
|---|---|---|---|---|---|---|---|
| Q180 Doctors use too many medications | ◯ | ◯ | ◯ | ◯ | ◯ | ◯ | ◯ |
| Q190 Doctors place too much trust in medications | ◯ | ◯ | ◯ | ◯ | ◯ | ◯ | ◯ |
| Q200 If doctors had more time with patients they would prescribe fewer medications | ◯ | ◯ | ◯ | ◯ | ◯ | ◯ | ◯ |

**Barriers to Meds**

| | Yes | No | NA - Not taking ANY medications | DON'T KNOW | REFUSED |
|---|---|---|---|---|---|
| Q210 In the past month, have you been worried about the cost of one or more of your medications? | ◯ | ◯ | ◯ | ◯ | ◯ |
| Q220 During the last month, did you ever skip doses of a medication or take a smaller dose to make the medication last longer, because you were concerned about the cost? | ◯ | ◯ | ◯ | ◯ | ◯ |

**Medication Adherence (Extent of Adherence, Voils et al)**

**The last questions I'm going to ask you about medications are about how often you take them. For one reason or another, many people can't or don't always take all of their pills the way their doctor or nurse as prescribed them. We want to know how often you have missed taking any of your blood pressure medications. Please rate your agreement with the following statements. Over the past 7 days, how often would you say...**
**(If participant is not taking blood pressure medications, omit the word blood pressure in each of the 3 questions. If participant not taking ANY medications, respond N/A to Q230 and skip to Q260)**

| | None of the time | A little of the time | Some of the time | Most of the time | All of the time | N/A - Not taking ANY medications | Don't know | REFUSED |
|---|---|---|---|---|---|---|---|---|
| Q230 I missed my blood pressure medication... | ◯ | ◯ | ◯ | ◯ | ◯ | ◯ | ◯ | ◯ |
| Q240 I skipped a dose of my blood pressure medication | ◯ | ◯ | ◯ | ◯ | ◯ | ◯ | ◯ | ◯ |
| Q250 I did not take a dose of my blood pressure medication | ◯ | ◯ | ◯ | ◯ | ◯ | ◯ | ◯ | ◯ |

**PROVIDER BEHAVIOR PCAS: Communication**

**Now I'd like to talk with you about your relationship with your main health care provider (doctor or nurse) that you see the most for your general medical care. How would you rate the following?**

| | Excellent | Very good | Good | Fair | Poor | Very Poor | DON'T KNOW | REFUSED |
|---|---|---|---|---|---|---|---|---|
| Q260 The level of detail of their questions about your symptoms and how you are feeling | ◯ | ◯ | ◯ | ◯ | ◯ | ◯ | ◯ | ◯ |
| Q270 Attention they give to what you have to say | ◯ | ◯ | ◯ | ◯ | ◯ | ◯ | ◯ | ◯ |
| Q280 Your regular doctor or nurse's explanations of your health problems or treatments that you need | ◯ | ◯ | ◯ | ◯ | ◯ | ◯ | ◯ | ◯ |
| Q290 Their instructions about symptoms to report and when to seek further care | ◯ | ◯ | ◯ | ◯ | ◯ | ◯ | ◯ | ◯ |

| | | | | | | | | |
|---|---|---|---|---|---|---|---|---|
| Q300 Their advice and help in making decisions about your care | ◯ | ◯ | ◯ | ◯ | ◯ | ◯ | ◯ | ◯ |

Q310 How often do you leave your doctor's office with unanswered questions?

◯ Always
◯ Almost Always
◯ A lot of the time
◯ Some of the time
◯ Almost Never
◯ Never
◯ DON'T KNOW
◯ REFUSED

**Trust and satisfaction with doctors PCAS (Trust)**

**Now please tell us how much you agree or disagree with the following things about your relationship with your primary doctor or nurse.**

| | Strongly Agree | Agree | Neither Agree Nor Disagree | Disagree | Strongly Disagree | DON'T KNOW | REFUSED |
|---|---|---|---|---|---|---|---|
| Q320 I can tell my doctor or nurse anything, even things that I might not tell anyone else | ◯ | ◯ | ◯ | ◯ | ◯ | ◯ | ◯ |
| Q330 My doctor or nurse sometimes pretends to know things when he/she is not really sure | ◯ | ◯ | ◯ | ◯ | ◯ | ◯ | ◯ |
| Q340 I completely trust my doctor or nurse's judgments about my medical care | ◯ | ◯ | ◯ | ◯ | ◯ | ◯ | ◯ |
| Q350 My doctor or nurse cares more about holding down costs than about doing what's needed for my health | ◯ | ◯ | ◯ | ◯ | ◯ | ◯ | ◯ |
| Q360 My doctor would always tell me the truth about my health even if there's bad news | ◯ | ◯ | ◯ | ◯ | ◯ | ◯ | ◯ |
| Q370 My doctor or nurse cares as much as I do about my health | ◯ | ◯ | ◯ | ◯ | ◯ | ◯ | ◯ |
| Q380 If a mistake was made in my treatment, he/she would try to hide it from me | ◯ | ◯ | ◯ | ◯ | ◯ | ◯ | ◯ |

Q390 All things considered, how much do you trust your primary doctor or nurse? We'd like you to rate this on a scale from 1-10, where 1 means you don't' trust them at all and 10 means you trust them completely.

○ 1 ○ 2 ○ 3 ○ 4 ○ 5 ○ 6 ○ 7 ○ 8 ○ 9 ○ 10

## Perceived Racism (LaVeist )

**Please tell me how much you agree or disagree with the following statements:**

| | Strongly Agree | Agree | Neither Agree Nor Disagree | Disagree | Strongly Disagree | DON'T KNOW | REFUSED |
|---|---|---|---|---|---|---|---|
| Q400 Doctors treat African American/Black and white people the same | ○ | ○ | ○ | ○ | ○ | ○ | ○ |
| Q410 Racial discrimination in a doctor's office is common | ○ | ○ | ○ | ○ | ○ | ○ | ○ |
| Q420 African Americans/Blacks and Whites receive the same kind of care in hospitals | ○ | ○ | ○ | ○ | ○ | ○ | ○ |
| Q430 African Americans/Blacks can get the care they want as equally as white people can | ○ | ○ | ○ | ○ | ○ | ○ | ○ |
| Q440 Doctors will discriminate against people who have a genetic risk for kidney disease | ○ | ○ | ○ | ○ | ○ | ○ | ○ |

## PATIENT ACTIVATION (PAM)

**Below are some statements that people sometimes make when they talk about their health. Please tell me how much you agree or disagree with each statement. Your answers should be what is true for you and not just what you your doctors or nurses want you to say.**

| | Strongly Agree | Agree | Disagree | Strongly Disagree | DON'T KNOW | REFUSED |
|---|---|---|---|---|---|---|
| Q450 When all is said and done, I am the person who is responsible for taking care of my health | ○ | ○ | ○ | ○ | ○ | ○ |
| Q460 Taking an active role in my own health care is the most important thing that affects my health | ○ | ○ | ○ | ○ | ○ | ○ |

| | | | | | | |
|---|---|---|---|---|---|---|
| Q470 I am confident I can help prevent or reduce problems associated with my health | ◯ | ◯ | ◯ | ◯ | ◯ | ◯ |

Q480 I know what each of my prescribed medications do

- ◯ Strongly Agree
- ◯ Agree
- ◯ Disagree
- ◯ Strongly Disagree
- ◯ DON'T KNOW
- ◯ REFUSED
- ◯ Not taking any medications

| | Strongly Agree | Agree | Disagree | Strongly Disagree | DON'T KNOW | REFUSED |
|---|---|---|---|---|---|---|
| Q490 I am confident that I can tell whether I need to go to the doctor or whether I can take care of a health problem myself | ◯ | ◯ | ◯ | ◯ | ◯ | ◯ |
| Q500 I am confident that I can tell my doctor concerns that I have, even when he or she does not ask. | ◯ | ◯ | ◯ | ◯ | ◯ | ◯ |
| Q510 I am confident that I can follow through on medical treatments I may need to do at home | ◯ | ◯ | ◯ | ◯ | ◯ | ◯ |
| Q520 I understand my health problems and what causes them | ◯ | ◯ | ◯ | ◯ | ◯ | ◯ |
| Q530 I know what treatments are available for my health problems | ◯ | ◯ | ◯ | ◯ | ◯ | ◯ |
| Q540 I have been able to maintain (keep up with) lifestyle changes, like eating right or exercising | ◯ | ◯ | ◯ | ◯ | ◯ | ◯ |
| Q550 I know how to prevent problems with my health | ◯ | ◯ | ◯ | ◯ | ◯ | ◯ |
| Q560 I am confident that if I get new health problems, I can figure out what to do about them | ◯ | ◯ | ◯ | ◯ | ◯ | ◯ |
| Q570 I am confident that I can keep lifestyle changes, like eating right and exercising, even during times of stress | ◯ | ◯ | ◯ | ◯ | ◯ | ◯ |

Q580 How confident are you filling out forms by yourself?

◯ Not at all confident
◯ A little bit confident
◯ Somewhat confident
◯ Quite a bit confident
◯ Very much confident
◯ DON'T KNOW
◯ REFUSED

## Now we'd like to ask you some questions about kidney problems.

Q590 Do you have a family member who has had kidney problems?

◯ Yes
◯ No
◯ DON'T KNOW
◯ REFUSED

Q600 Do you have a person close to you (like a friend) who has had kidney problems?

◯ Yes
◯ No
◯ DON'T KNOW
◯ REFUSED

Q610 Do you have any kidney problems?

◯ Yes
◯ No (GO TO Q630)
◯ DON'T KNOW
◯ REFUSED

Q620 Is your kidney problem so serious that you are on dialysis?

◯ Yes
◯ No
◯ DON'T KNOW
◯ REFUSED

## Modified IPQ- Kidney disease

| | Not at all | A little bit | Somewhat | Very much | Extremely | DON'T KNOW | REFUSED |
|---|---|---|---|---|---|---|---|
| Q630 How concerned are you about getting kidney problems? | ◯ | ◯ | ◯ | ◯ | ◯ | ◯ | ◯ |
| Q640 How much do you think you can prevent getting kidney problems? | ◯ | ◯ | ◯ | ◯ | ◯ | ◯ | ◯ |
| Q650 Do you understand what you can do to get checked for kidney problems? | ◯ | ◯ | ◯ | ◯ | ◯ | ◯ | ◯ |

| | | | | | | | |
|---|---|---|---|---|---|---|---|
| Q660 Do you understand what you can do to prevent getting kidney problems? | ◯ | ◯ | ◯ | ◯ | ◯ | ◯ | ◯ |
| Q670 How much do you think you can tell, or feel if you had kidney problems? | ◯ | ◯ | ◯ | ◯ | ◯ | ◯ | ◯ |
| Q680 If you had kidney problems, how much do you think medical treatments or medications could keep the problems from getting worse? | ◯ | ◯ | ◯ | ◯ | ◯ | ◯ | ◯ |

---

Q690 How much do you think that kidney problems can be caused by high blood pressure?

◯ Not at all
◯ A little bit
◯ Somewhat
◯ Very much
◯ Extremely
◯ DON'T KNOW
◯ REFUSED

---

Q700 Has your doctor explained to you that kidney problems can be caused by high blood pressure?

◯ Yes
◯ No
◯ DON'T KNOW
◯ REFUSED

---

Q710 How much do you think that people inherit kidney problems through their genes?

◯ Not at all
◯ A little bit
◯ Somewhat
◯ Very much
◯ Extremely
◯ DON'T KNOW
◯ REFUSED

**Now I'd like to ask you some questions about genes and genetics and ways things may be passed down through generations.**

Q720 How well do you understand the relationship between genetics and health?

◯ Not at all
◯ A little bit
◯ Somewhat
◯ Very much
◯ Extremely
◯ DON'T KNOW
◯ REFUSED

Q730 Have you ever had a genetic test?

- ◯ Yes
- ◯ No
- ◯ DON'T KNOW
- ◯ REFUSED

## Beliefs toward genetic testing

| | Not at all | A little bit | Somewhat | Very much | Extremely | DON'T KNOW | REFUSED |
|---|---|---|---|---|---|---|---|
| Q740 How well do you understand genetic testing? | ◯ | ◯ | ◯ | ◯ | ◯ | ◯ | ◯ |
| Q750 In general, do you think it's a good idea to get genetic testing to find out about if you are at risk for getting a common disease like high blood pressure, diabetes or kidney disease | ◯ | ◯ | ◯ | ◯ | ◯ | ◯ | ◯ |
| Q760 How much do you think that differences in people's genes affect their risk for these kinds of common diseases? | ◯ | ◯ | ◯ | ◯ | ◯ | ◯ | ◯ |
| Q770 Do you think African American or Black people are more likely to have diseases like high blood pressure, diabetes and kidney disease than European American or White people? | ◯ | ◯ | ◯ | ◯ | ◯ | ◯ | ◯ |
| Q780 Do you think these differences in health are because of differences in their genes? | ◯ | ◯ | ◯ | ◯ | ◯ | ◯ | ◯ |
| Q790 Do you think these differences in health are because of differences in their behaviors or culture? | ◯ | ◯ | ◯ | ◯ | ◯ | ◯ | ◯ |
| Q800 Do you think these differences in health are because of differences in the amount of stress Black and White people have? | ◯ | ◯ | ◯ | ◯ | ◯ | ◯ | ◯ |

Q810 Do you think these differences in health are because of differences in their environments, like where they live, their access to care, or pollution? ◯ ◯ ◯ ◯ ◯ ◯ ◯

---

Q820 Let's say your doctors thought your risk of getting a disease as a Black/African American person was based on differences in genes, rather than your behavior or your environment. How do you think this would change the way they view you?

◯ It would not change anything
◯ They would have a more negative view of me
◯ They would have a more positive view of me
◯ DON'T KNOW
◯ REFUSED

## Knowledge /Beliefs toward APOL1 testing

**You have agreed to get tested for changes in the APOL1 gene that may lead to increased risk of kidney problems or kidney disease.**

| | Not at all | A little bit | Somewhat | Very much | Extremely | DON'T KNOW | REFUSED |
|---|---|---|---|---|---|---|---|
| Q830 How well do you understand APOL1 genetic testing? | ◯ | ◯ | ◯ | ◯ | ◯ | ◯ | ◯ |

---

Q840 If it were available, how much would you want your children do get tested for changes in the APOL1 gene?

◯ Not at all
◯ A little bit
◯ Somewhat
◯ Very much
◯ Extremely
◯ NA- no children
◯ DON'T KNOW
◯ REFUSED

Q850 What would you do if you received a result from the APOL1 genetic testing that showed you had a higher risk for kidney disease? (DON'T READ OPTIONS)

- ☐ Nothing
- ☐ Tell a friend/relative
- ☐ Be upset/worry
- ☐ Talk to or go to a doctor/specialist; seek treatment
- ☐ Increase physical activity
- ☐ Change diet
- ☐ Drink more water
- ☐ Take blood pressure medications more
- ☐ Stop taking blood pressure medications
- ☐ Reduce/quit drinking
- ☐ Quit smoking
- ☐ Other:
- ☐ Research/get more information about the APOL1 gene and/or kidney disease
- ☐ Pray or meditate
- ☐ Take action to be healthier/change lifestyle (not specific)
- ☐ Be positive/try to be positive; not stress test result, information is power
- ☐ Try to lose weight
- ☐ Reduce life stress/take it easy (in general)
- ☐ Talk to genetic counselor
- ☐ Use alternative/home remedies
- ☐ Learn more about one's family history and possibility of passing onto children
- ☐ Look into health/life insurance coverage
- ☐ Sleep/rest more
- ☐ DON'T KNOW
- ☐ REFUSED

Other, specify: ______________________________

| | Not at all | A little bit | Somewhat | Very much | Extremely | DON'T KNOW | REFUSED |
|---|---|---|---|---|---|---|---|
| Q860 Do you think there would be medical treatments to keep you from getting kidney disease or kidney problems? | ◯ | ◯ | ◯ | ◯ | ◯ | ◯ | ◯ |
| Q870 Would you be upset about having a test that showed you were at higher risk for kidney disease? | ◯ | ◯ | ◯ | ◯ | ◯ | ◯ | ◯ |
| Q880 Would you be worried that your test results might be given to someone else without your permission? | ◯ | ◯ | ◯ | ◯ | ◯ | ◯ | ◯ |

Q890 If the genetic test shows you DID have an increased risk for kidney disease, do you think it would change how often you take the blood pressure medications your doctor or nurse has prescribed for you?

- ◯ Yes
- ◯ No (Skip to 910)
- ◯ DON'T KNOW
- ◯ REFUSED

Q900 Would you take your blood pressure medications more often, less often, or the way you take them now? (If pt says they don't take them, ask if they think they would start to)

◯ More often (or would you start to take them)
◯ Less often
◯ The way I take them now.
◯ DON'T KNOW
◯ REFUSED

Q910 Now please think about how you would feel if the result from APOL1 genetic testing showed you DID NOT have an increased risk for kidney disease. Would it change how often you take the blood pressure medications your doctor or nurse has prescribed for you?

◯ Yes
◯ No (Skip to 1270)
◯ DON'T KNOW
◯ REFUSED

Q920 Do you think you would take your blood pressure medications more often, less often, or the way you take them now? (If pt says they don't take them, ask if they think they would start to)

◯ More often (or will you start to take them)
◯ Less often
◯ The way I take them now.
◯ DON'T KNOW
◯ REFUSED

**Depression (PHQ8)**

**Now we'd like to ask you about your mood. Over the past 2 weeks, how often have you been bothered by any of the following problems:**

| | Not at all | Several days | More than half the days | Nearly everyday | DON'T KNOW | REFUSED |
|---|---|---|---|---|---|---|
| Q1270 Little interest or pleasure in doing things | ◯ | ◯ | ◯ | ◯ | ◯ | ◯ |
| Q1280 Feeling down, depressed, or hopeless | ◯ | ◯ | ◯ | ◯ | ◯ | ◯ |
| Q1290 Trouble falling or staying asleep, or sleeping too much | ◯ | ◯ | ◯ | ◯ | ◯ | ◯ |
| Q1300 Feeling tired or having little energy | ◯ | ◯ | ◯ | ◯ | ◯ | ◯ |
| Q1310 Poor appetite or overeating | ◯ | ◯ | ◯ | ◯ | ◯ | ◯ |
| Q1320 Feeling bad about yourself, or that you are a failure or have let yourself or your family down | ◯ | ◯ | ◯ | ◯ | ◯ | ◯ |

| | | | | | | |
|---|---|---|---|---|---|---|
| Q1330 Trouble concentrating on things, such as reading the newspaper or watching television | ○ | ○ | ○ | ○ | ○ | ○ |
| Q1340 Moving or speaking so slowly that other people could have noticed-- or the opposite - being so fidgety or restless that you have been moving around a lot more than usual. | ○ | ○ | ○ | ○ | ○ | ○ |

## GAD-2 (Generalized Anxiety)

**Over the last two weeks, how often have you been bothered by the following problems?**

| | Not at all | Several days | More than half the days | Nearly everyday | DON'T KNOW | REFUSED |
|---|---|---|---|---|---|---|
| Q1350 Feeling nervous, anxious or on edge | ○ | ○ | ○ | ○ | ○ | ○ |
| Q1360 Not being able to sleep or control worrying? | ○ | ○ | ○ | ○ | ○ | ○ |

## Perceived Stress Scale

| | Never | Almost Never | Sometimes | Fairly Often | Very Often | DON'T KNOW | REFUSED |
|---|---|---|---|---|---|---|---|
| Q1370 In the last month, how often have you felt that you were not able to control the important things in your life? | ○ | ○ | ○ | ○ | ○ | ○ | ○ |
| Q1380 In the last month, how often have you felt confident about your ability to handle your personal problems? | ○ | ○ | ○ | ○ | ○ | ○ | ○ |
| Q1390 In the last month, how often have you felt that things were going your way | ○ | ○ | ○ | ○ | ○ | ○ | ○ |
| Q1400 In the last month, how often have you felt difficulties were piling up so high that you could not overcome them? | ○ | ○ | ○ | ○ | ○ | ○ | ○ |

## Social Support (MOSS- 6)

**People sometimes look to others for companionship, assistance or other types of support. How often is each of the following kinds of support available to you if you need it?**

| | None of the time | A little of the time | Some of the time | Most of the time | All of the time | DON'T KNOW | REFUSED |
|---|---|---|---|---|---|---|---|

| | | | | | | | |
|---|---|---|---|---|---|---|---|
| Q1410 Someone to share your most private worries and fears with | ○ | ○ | ○ | ○ | ○ | ○ | ○ |
| Q1420 Someone to turn to for suggestions about how to deal with a personal problem: | ○ | ○ | ○ | ○ | ○ | ○ | ○ |
| Q1430 Someone to help you if you were confined to bed | ○ | ○ | ○ | ○ | ○ | ○ | ○ |
| Q1440 Someone to take you to the doctor if you needed it | ○ | ○ | ○ | ○ | ○ | ○ | ○ |
| Q1450 Someone to love and make you feel wanted | ○ | ○ | ○ | ○ | ○ | ○ | ○ |
| Q1460 Someone to do something enjoyable with | ○ | ○ | ○ | ○ | ○ | ○ | ○ |

## Life Chaos

**For the next set questions, we'd like you to think about the things going on in your life. Tell me how much you agree or disagree with each statement.**

| | Strongly Agree | Agree | Neither Agee Nor Disagree | Disagree | Strongly Disagree | DON'T KNOW | REFUSED |
|---|---|---|---|---|---|---|---|
| Q1470 My life is organized | ○ | ○ | ○ | ○ | ○ | ○ | ○ |
| Q1480 My life is unstable | ○ | ○ | ○ | ○ | ○ | ○ | ○ |
| Q1490 My routine is the same from week to week | ○ | ○ | ○ | ○ | ○ | ○ | ○ |
| Q1500 My daily activities from week to week are unpredictable | ○ | ○ | ○ | ○ | ○ | ○ | ○ |
| Q1510 Keeping a schedule is difficult for me | ○ | ○ | ○ | ○ | ○ | ○ | ○ |
| Q1520 I do not like making appointments too far in advance because I do not know what may come up | ○ | ○ | ○ | ○ | ○ | ○ | ○ |

Q1530 Now I'd like to ask you some questions about your lifestyle. During the past 7 days, how much physical activity (like walking) or exercise did you do?

○ Less than an hour
○ 1 to 1.5 hours
○ 1.5 to 2 hours
○ More than 2 hours
○ DON'T KNOW
○ REFUSED

Q1540 Have you become more physically active or exercised more in the past 6 months?

◯ Yes
◯ No
◯ DON'T KNOW
◯ REFUSED

Q1550 In general, do you eat a healthy diet?

◯ Not at all
◯ A little bit
◯ Somewhat
◯ Very much
◯ Extremely
◯ DON'T KNOW
◯ REFUSED

Q1560 Have you made changes to your diet in the past 6 months?

◯ Yes
◯ No
◯ DON'T KNOW
◯ REFUSED

Q1570 Now we'd like to ask you about some of your other health habits: In the past month, did you smoke or chew tobacco?

◯ Yes
◯ No (GO TO Q1600)
◯ DON'T KNOW
◯ REFUSED

Q1580 How many cigarettes (or cigars) on average do you smoke each day?

__________________________________
(If < 1, write '0'. If participant doesn't know, write "888")

Q1590 Do you currently want to quit smoking?

◯ Yes
◯ No
◯ DON'T KNOW
◯ REFUSED

Q1600 How often do you have a drink containing alcohol?

◯ Never (GO TO Q1630)
◯ Monthly or less
◯ 2 to 4 times a month
◯ 2 to 3 times a week
◯ 4 or more times a week
◯ DON'T KNOW
◯ REFUSED

Q1610 How many drinks containing alcohol do you have on a typical day when you are drinking? (DON'T READ OPTIONS)

◯ 1 None
◯ 1 or 2
◯ 3 or 4
◯ 5 or 6
◯ 7 to 9
◯ 10 or more
◯ DON'T KNOW
◯ REFUSED

Q1620 How often do you have 6 or more drinks on one occasion?

◯ Never
◯ Less than monthly
◯ Monthly
◯ Weekly
◯ Daily
◯ DON'T KNOW
◯ REFUSED

**Co-morbidities (Charlson Comorbidity Index)**

**Now we'd like to ask you questions about other medical problems you may have:**

Q1630 Have you ever had a heart attack?

◯ Yes
◯ No
◯ DON'T KNOW
◯ REFUSED

Q1640 Have you ever been hospitalized or treated for heart failure? (if participant doesn't understand heart failure, add: "you may have felt more short of breath and the doctor may have told you that you had fluid in your lungs or that your heart was not working properly.")

◯ Yes
◯ No
◯ DON'T KNOW
◯ REFUSED

Q 1650 Do you have chronic bronchitis or emphysema?  (If a patient doesn't understand these words, add: "Do you cough first thing in the morning in winter, and do you cough up mucus on most of these days?")

◯ Yes
◯ No
◯ DON'T KNOW
◯ REFUSED

Q 1660 Do you have asthma?

◯ Yes
◯ No
◯ DON'T KNOW
◯ REFUSED

Q1670 Have you ever had an operation to improve the flow of blood in the arteries in your legs or been told that you had blockages in the arteries inside your legs?

◯ Yes
◯ No
◯ DON'T KNOW
◯ REFUSED

Q1680 Do you ever get pains in the muscles in the backs of your legs when you are walking, especially walking uphill or hurrying, making it necessary to stop or slow down?

◯ Yes
◯ No (GO TO 1700)
◯ DON'T KNOW
◯ REFUSED

Q1690 Is it relieved when you stop walking or slow down in less than 10 minutes?

◯ Yes
◯ No
◯ DON'T KNOW
◯ REFUSED

Q1700 Do you have diabetes or high blood sugar?

◯ Yes
◯ No (GO TO Q1720)
◯ DON'T KNOW
◯ REFUSED

Q1710 Has diabetes caused problems with your kidneys, your eyes or with the feeling in your feet and legs?

◯ Yes
◯ No
◯ DON'T KNOW
◯ REFUSED

Q1720 Do you have trouble with your liver?

◯ Yes
◯ No (GO TO Q1740)
◯ DON'T KNOW
◯ REFUSED

Q1730 Do you have cirrhosis of the liver or permanent liver damage?

◯ Yes
◯ No
◯ DON'T KNOW
◯ REFUSED

Q1740 Have you had trouble with stomach ulcers?

◯ Yes
◯ No
◯ DON'T KNOW
◯ REFUSED

Q1750 Have you been diagnosed with cancer in the last 5 years (except skin cancer)?

◯ Yes
◯ No (GO TO Q1780)
◯ DON'T KNOW
◯ REFUSED

Q1760 What type of cancer was it? Specify:

_______________________________

Q1770 Did it spread to other parts of the body?

◯ Yes
◯ No
◯ DON'T KNOW
◯ REFUSED

Q1780 Do you have any rheumatalogic diseases? (check yes if one of the following: Rheumatoid Arthritis, Psoriatic Arthritis, Lupus Polymyositis, Mixed connective tissue disease, Polymyalgia rheumatica/temporal arthritis. DO NOT check if patient only has osteoarthritis)

◯ Yes
◯ No
◯ DON'T KNOW
◯ REFUSED

Specify:

_______________________________

**Now, we'd like to ask you some questions about your medical care.**

Q1790 Overall, how difficult is it for you to get medical care when you need it?

◯ Not at all difficult
◯ A little bit difficult
◯ Somewhat difficult
◯ Very much difficult
◯ Extremely difficult
◯ DON'T KNOW
◯ REFUSED

1800 Do you currently have a person you consider your primary care doctor or primary care nurse?

◯ Yes
◯ No
◯ DON'T KNOW
◯ REFUSED

Q1810 In the last 6 months, how many times did you see any primary care doctor? (If none, enter '0')

_______________________________
(For Don't know type: 888; For Refused, type:999)

Q1820 On average, how long does it take you to get to your doctor's office from your home?  (Enter number of MINUTES)

_______________________________
(For Don't know type: 888; For Refused, type:999)

Q1830 On average, how much time do you spend at your primary care doctor's office? Please include both the time you wait to see your doctor and the time the doctor spends with you. (Enter number of MINUTES)

__________________________________
(For Don't know type: 888; For Refused, type:999)

Q1840 What form of transportation do you usually use to get to your primary care doctor's office?

◯ Public Transportation
◯ Ambulette/Access-a-Ride
◯ Friend or family drives me
◯ Taxi
◯ Walking
◯ Other
◯ I drive myself
◯ DON'T KNOW
◯ REFUSED

Specify: __________________________________

Q1850 In the last 6 months, how many times did you visit specialist doctors? That is, how many visits did you go to doctors other than your primary care doctor such as a heart or kidney doctor?  (Enter # of times. For none, enter '0')

__________________________________
(For Don't know type: 888; For Refused, type:999)

Q1860 In the last 6 months, how many times did you go to an emergency room? (Enter # of times. For none, enter '0')

__________________________________
(For Don't know type: 888; For Refused, type:999)

Q1870 In the last 6 months, how many times were you admitted to the hospital? That is, how many times did you spend more than 24 hours in the hospital and not just in the emergency room?  (Enter # of times. For None, enter '0')

__________________________________
(For Don't know type: 888; For Refused, type:999)

Q1880 In the past six months, how many times did you see a mental health provider, such as a psychologist, psychiatrist, or social worker? (Enter # of times. For none, enter '0')

__________________________________
(For Don't know type: 888; For Refused, type:999)

## Finally, we'd like to ask you a few questions about you!

Q1890 What is your marital status?

◯ Currently married
◯ Widowed
◯ Divorced
◯ Separated
◯ Part of an unmarried couple
◯ Never married
◯ DON'T KNOW
◯ REFUSED

Q1900 How many people are currently living in your household, including yourself? (Enter # of people)

__________________________________
(For Don't know type: 888; For Refused, type:999)

Q1910 Have you lived in the United States since birth?

◯ Yes
◯ No

Specify # of years living in the US: __________________________________

Q1920 What one thing were you doing most often during the past 4 weeks?

◯ Working full-time/part-time
◯ Looking for work/laid off
◯ Unable to work
◯ Going to school
◯ Retired
◯ Other
◯ DON'T KNOW
◯ REFUSED

Q1930 Do you consider yourself (CHOOSE ALL THAT APPLY):

☐ White
☐ Black or African American
☐ American Indian or Native American
☐ Asian or Pacific Islander
☐ Hispanic/Latino/Spanish
☐ Other
☐ DON'T KNOW
☐ REFUSED

Specify:

__________________________________

Q1940 And do you identify as:

◯ Puerto Rican
◯ Mexican
◯ Dominican
◯ Cuban
◯ Ecuadorian
◯ Other Latin American Identity
◯ None of the above
◯ DON'T KNOW
◯ REFUSED

Q1950 What kind of Health Insurance, if any, do you have right now? (CHOOSE ALL THAT APPLY)

☐ No Insurance/Sliding Scale
☐ Medicaid (SKIP TO Q1970)
☐ Medicare (SKIP TO Q1970)
☐ Private Health Insurance (SKIP TO Q1970)
☐ Military Health Care/VA (SKIP TO Q1970)
☐ Other Insurance (specify) (SKIP TO Q1970)
☐ DON'T KNOW
☐ REFUSED

Other, specify:

__________________________________

Q1960 (IF NO INSURANCE for Q 1950) How long has it been since you last had health coverage?

◯ 6 months or less
◯ More than 6 months, up to and including 1 year
◯ More than 1 year
◯ DON'T KNOW
◯ REFUSED

Q1970 Are your prescription medications covered (at least partially) by some kind of health insurance?

◯ Yes
◯ No
◯ DON'T KNOW
◯ REFUSED

Q1980 What was the last year of school you completed?

◯ Less than junior high or eighth grade (0-8 years of formal education)
◯ Less than high school (8-11 years)
◯ High school graduate or GED (12 years)
◯ Some college/trade or technical school (13-15 years)
◯ College graduate/professional training (16 or more years)
◯ DON'T KNOW
◯ REFUSED

Q1990 In which range was the total income of your household last year before taxes? Please include all the money that you received from any source.

◯ Less than $5000 a year, or less than $417 per month
◯ $5000 up to $15,000 a year, or $417 to $1250 per month
◯ $15,000 up to $30,000 a year, or $1,250 to $2,500 per month
◯ $30,000 up to $45,000 a year, or $2,500 to $3,750 per month
◯ $45,000 up to $60,000 a year, or $3,750 to $5,000 per month
◯ $60,000 or more a year, or $5,000 or more per month
◯ DON'T KNOW
◯ REFUSED

Thank you, you have finished the survey!

# 12 Months Survey

Record ID ______________________

Research history collected:
Recruitment: [baseline_arm_1][research_history]
Survey Baseline: [baseline_arm_1][research_history_baseline]
Survey 3 mo: [followup_3_mo_arm_1][research_history_3m]
Call 9 mo: [followup_12_mo_arm_1][research_history_9_mo]
Survey 12 mo: [followup_12_mo_arm_1][research_history12m]

If 'Yes/No' in any of the categories above, skip 1st question.

1. Have you ever taken part in another research study other than this one?
- ◯ Yes
- ◯ No

## Quality of Life (SF-1)

10 Would you say your health in general is excellent, very good, good, fair, poor or very poor? (CHOOSE ONE ONLY)

- ◯ Excellent
- ◯ Very good
- ◯ Good
- ◯ Fair
- ◯ Poor
- ◯ Very poor
- ◯ DON'T KNOW
- ◯ REFUSED

## Modified from Brief IPQ -HTN

30 How much does having high blood pressure affect your life? (CHOOSE ONE ONLY)

- ◯ Not at all
- ◯ A little bit
- ◯ Somewhat
- ◯ Very much
- ◯ Extremely
- ◯ DON'T KNOW
- ◯ REFUSED

40 How much control do you feel you have over your high blood pressure? (CHOOSE ONE ONLY)

- ◯ Not at all
- ◯ A little bit
- ◯ Somewhat
- ◯ Very much
- ◯ Extremely
- ◯ DON'T KNOW
- ◯ REFUSED

50 How much do you think your treatment can help your high blood pressure? (CHOOSE ONE ONLY)

- ◯ Not at all
- ◯ A little bit
- ◯ Somewhat
- ◯ Very much
- ◯ Extremely
- ◯ DON'T KNOW
- ◯ REFUSED

60 How concerned are you about your high blood pressure? (CHOOSE ONE ONLY)

- ◯ Not at all
- ◯ A little bit
- ◯ Somewhat
- ◯ Very much
- ◯ Extremely
- ◯ DON'T KNOW
- ◯ REFUSED

70 How much do you think that people inherit high blood pressure through their genes? (CHOOSE ONE ONLY)

- ◯ Not at all
- ◯ A little bit
- ◯ Somewhat
- ◯ Very much
- ◯ Extremely
- ◯ DON'T KNOW
- ◯ REFUSED

Now we would like to ask you some questions about
medications you have been given or that you take.

75 Are you currently taking medications for your blood pressure or has a provider told you that you need to take OR SHOULD take them? (CHOOSE ONE ONLY)

- ◯ Yes, I am taking blood pressure medications
- ◯ No. I have been told I SHOULD take them but I am not currently taking them.
- ◯ No. I have not been told to take blood pressure medications.

**Outcomes Expectations- HTN (Adapted from the CAATCH Study)**
**Please let me know how much you agree or disagree with the following (If participant not taking blood pressure medications because they have not been prescribed to him/her: Please answer the following questions thinking about how you would feel if you were told you needed to take blood pressure medications):**

| | Strongly Agree | Agree | Neither Agree Nor Disagree | Disagree | Strongly Disagree | DON'T KNOW | REFUSED |
|---|---|---|---|---|---|---|---|

| | | | | | | | |
|---|---|---|---|---|---|---|---|
| 80 I (would) worry about the long-term effects of my blood pressure medications. (CHOOSE ONE ONLY) | ○ | ○ | ○ | ○ | ○ | ○ | ○ |
| 90 I (would) expect medication to control my high blood pressure (CHOOSE ONE ONLY) | ○ | ○ | ○ | ○ | ○ | ○ | ○ |
| 100 I will (would) not have a stroke or heart attack if I take (took) blood pressure medication (CHOOSE ONE ONLY) | ○ | ○ | ○ | ○ | ○ | ○ | ○ |
| 110 I will (would) not have kidney problems or kidney failure if I take (took) blood pressure medication (CHOOSE ONE ONLY) | ○ | ○ | ○ | ○ | ○ | ○ | ○ |
| 120 I (would) expect to take blood pressure medication for the rest of my life (CHOOSE ONE ONLY) | ○ | ○ | ○ | ○ | ○ | ○ | ○ |
| 130 I (would) take my blood pressure medication only when I have symptoms (CHOOSE ONE ONLY) | ○ | ○ | ○ | ○ | ○ | ○ | ○ |
| 140 The medication (would help) helps me reduce stress (CHOOSE ONE ONLY) | ○ | ○ | ○ | ○ | ○ | ○ | ○ |
| 150 The blood pressure medication will help (would help) me live longer (CHOOSE ONE ONLY) | ○ | ○ | ○ | ○ | ○ | ○ | ○ |
| 160 I will (would) feel better if I take (took) blood pressure medication (CHOOSE ONE ONLY) | ○ | ○ | ○ | ○ | ○ | ○ | ○ |
| 170 I will (would ) not need medication anymore when my blood pressure is normal (CHOOSE ONE ONLY) | ○ | ○ | ○ | ○ | ○ | ○ | ○ |

## Barriers to Meds

| | Yes | No | N/A - Not taking ANY medications | DON'T KNOW | REFUSED |
|---|---|---|---|---|---|
| 210 In the past month, have you been worried about the cost of one or more of your medications? (CHOOSE ONE ONLY) | ○ | ○ | ○ | ○ | ○ |
| 220 During the last month, did you ever skip doses of a medication or take a smaller dose to make the medication last longer, because you were concerned about the cost? (CHOOSE ONE ONLY) | ○ | ○ | ○ | ○ | ○ |

## Medication Adherence (Extent of Adherence, Voils et al.)

**The last questions I'm going to ask you about medications are about how often you take them. For one reason or another, many people can't or don't always take all of their pills the way their doctor or nurse prescribed them. We want to know how often you have missed taking any of your blood pressure medications.(If participant is not taking blood pressure medications, omit the word blood pressure in each of the 3 questions. If participant not taking ANY medications, respond N/A to Q230 and skip to Q260).**

**Over the past 7 days, how often would you say...**

| | None of the time | A little of the time | Some of the time | Most of the time | All of the time | N/A - Not taking ANY medications (Skip to q260) | DON'T KNOW | REFUSED |
|---|---|---|---|---|---|---|---|---|
| 230 I missed my (blood pressure) medication. | ○ | ○ | ○ | ○ | ○ | ○ | ○ | ○ |
| 240 I skipped a dose of my (blood pressure) medication. | ○ | ○ | ○ | ○ | ○ | ○ | ○ | ○ |
| 250 I did not take a dose of my (blood pressure) medication. | ○ | ○ | ○ | ○ | ○ | ○ | ○ | ○ |

**Trust and satisfaction with doctors PCAS (Trust)**

**Now please tell me how much you agree or disagree with the following statements about your relationship with your primary doctor or nurse.**

| | Strongly Agree | Agree | Neither Agree Nor Disagree | Disagree | Strongly Disagree | DON'T KNOW | REFUSED |
|---|---|---|---|---|---|---|---|
| 320 I can tell my doctor or nurse anything, even things that I might not tell anyone else (CHOOSE ONE ONLY) | ○ | ○ | ○ | ○ | ○ | ○ | ○ |
| 330 My doctor or nurse sometimes pretends to know things when he/she is not really sure (CHOOSE ONE ONLY) | ○ | ○ | ○ | ○ | ○ | ○ | ○ |
| 340 I completely trust my doctor or nurse's judgments about my medical care (CHOOSE ONE ONLY) | ○ | ○ | ○ | ○ | ○ | ○ | ○ |
| 350 My doctor or nurse cares more about holding down costs than about doing what's needed for my health (CHOOSE ONE ONLY) | ○ | ○ | ○ | ○ | ○ | ○ | ○ |
| 360 My doctor would always tell me the truth about my health even if there's bad news (CHOOSE ONE ONLY) | ○ | ○ | ○ | ○ | ○ | ○ | ○ |
| 370 My doctor or nurse cares as much as I do about my health (CHOOSE ONE ONLY) | ○ | ○ | ○ | ○ | ○ | ○ | ○ |
| 380 If a mistake was made in my treatment, he/she would try to hide it from me (CHOOSE ONE ONLY) | ○ | ○ | ○ | ○ | ○ | ○ | ○ |

390 All things considered, how much do you trust your primary doctor or nurse? We'd like you to rate this on a scale from 1-10, where 1 means you don't' trust them at all and 10 means you trust them completely. (CHOOSE ONE ONLY)

○ 1 ○ 2 ○ 3 ○ 4 ○ 5 ○ 6 ○ 7 ○ 8 ○ 9 ○ 10

**Perceived Racism (LaVeist)**

**Please tell me how much you agree or disagree with the following statements:**

| | Strongly Agree | Agree | Neither Agree Nor Disagree | Disagree | Strongly Disagree | DON'T KNOW | REFUSED |
|---|---|---|---|---|---|---|---|
| 400 Doctors treat African American/Black and white people the same. (CHOOSE ONE ONLY) | ○ | ○ | ○ | ○ | ○ | ○ | ○ |
| 410 Racial discrimination in a doctor's office is common. (CHOOSE ONE ONLY) | ○ | ○ | ○ | ○ | ○ | ○ | ○ |
| 420 African Americans/Blacks and Whites receive the same kind of care in hospitals. (CHOOSE ONE ONLY) | ○ | ○ | ○ | ○ | ○ | ○ | ○ |
| 430 African Americans/Blacks can get the care they want as equally as white people can. (CHOOSE ONE | ○ | ○ | ○ | ○ | ○ | ○ | ○ |
| 440 Doctors will discriminate against people who have a genetic risk for kidney disease. | ○ | ○ | ○ | ○ | ○ | ○ | ○ |

**PATIENT ACTIVATION (PAM)**

**Below are some statements that people sometimes make when they talk about their health. Please tell me how much you agree or disagree with each statement. Your answers should be what is true for you and not just what you your doctors or nurses want you to say.**

| | Strongly Agree | Agree | Disagree | Strongly Disagree | DON'T KNOW | REFUSED |
|---|---|---|---|---|---|---|
| 450 When all is said and done, I am the person who is responsible for taking care of my health (CHOOSE ONE ONLY) | ○ | ○ | ○ | ○ | ○ | ○ |
| 460 Taking an active role in my own health care is the most important thing that affects my health (CHOOSE ONE ONLY) | ○ | ○ | ○ | ○ | ○ | ○ |
| 470 I am confident I can help prevent or reduce problems associated with my health. (CHOOSE ONE ONLY) | ○ | ○ | ○ | ○ | ○ | ○ |

480 I know what each of my prescribed medications do.

- ◯ Strongly Agree
- ◯ Agree
- ◯ Disagree
- ◯ Strongly Disagree
- ◯ N/A - Not taking ANY medications
- ◯ DON'T KNOW
- ◯ REFUSED

| | Strongly Agree | Agree | Disagree | Strongly Disagree | DON'T KNOW | REFUSED |
|---|---|---|---|---|---|---|
| 490 I am confident that I can tell whether I need to go to the doctor or whether I can take care of a health problem myself. (CHOOSE ONE ONLY) | ◯ | ◯ | ◯ | ◯ | ◯ | ◯ |
| 500 I am confident that I can tell my doctor concerns that I have, even when he or she does not ask. (CHOOSE ONE ONLY) | ◯ | ◯ | ◯ | ◯ | ◯ | ◯ |
| 510 I am confident that I can follow through on medical treatments I may need to do at home (CHOOSE ONE ONLY) | ◯ | ◯ | ◯ | ◯ | ◯ | ◯ |
| 520 I understand my health problems and what causes them. (CHOOSE ONE ONLY) | ◯ | ◯ | ◯ | ◯ | ◯ | ◯ |
| 530 I know what treatments are available for my health problems. (CHOOSE ONE ONLY) | ◯ | ◯ | ◯ | ◯ | ◯ | ◯ |
| 540 I have been able to maintain (keep up with) lifestyle changes, like eating right or exercising. (CHOOSE ONE ONLY) | ◯ | ◯ | ◯ | ◯ | ◯ | ◯ |
| 550 I know how to prevent problems with my health. (CHOOSE ONE ONLY) | ◯ | ◯ | ◯ | ◯ | ◯ | ◯ |
| 560 I am confident that if I get new health problems, I can figure out what to do about them. (CHOOSE ONE ONLY) | ◯ | ◯ | ◯ | ◯ | ◯ | ◯ |
| 570 I am confident that I can keep lifestyle changes, like eating right and exercising, even during times of stress. (CHOOSE ONE ONLY) | ◯ | ◯ | ◯ | ◯ | ◯ | ◯ |

Now we'd like to ask you some questions about kidney problems.

| | |
|---|---|
| 610 Do you have any kidney problems? (CHOOSE ONLY ONE) (from Charleson) | ◯ Yes<br>◯ No (GO TO Q630)<br>◯ DON'T KNOW<br>◯ REFUSE |
| 620 Is your kidney problem so serious that you are on dialysis? (CHOOSE ONLY ONE) (from Charleson) | ◯ Yes<br>◯ No<br>◯ DON'T KNOW<br>◯ REFUSE |

## Modified IPQ- Kidney disease

| | Not at all | A little bit | Somewhat | Very much | Extremely | DON'T KNOW | REFUSED |
|---|---|---|---|---|---|---|---|
| 630 How concerned are you about getting kidney problems? (CHOOSE ONE ONLY) | ◯ | ◯ | ◯ | ◯ | ◯ | ◯ | ◯ |
| 640 How much do you think you can prevent getting kidney problems? (CHOOSE ONE ONLY) | ◯ | ◯ | ◯ | ◯ | ◯ | ◯ | ◯ |
| 650 Do you understand what you can do to get checked for kidney problems? (Adapted from MICRA) | ◯ | ◯ | ◯ | ◯ | ◯ | ◯ | ◯ |
| 660 Do you understand what you can do to prevent getting kidney problems? (Adapted from MICRA) | ◯ | ◯ | ◯ | ◯ | ◯ | ◯ | ◯ |
| 670 How much do you think you can tell, or feel if you had kidney problems? (CHOOSE ONE ONLY) | ◯ | ◯ | ◯ | ◯ | ◯ | ◯ | ◯ |
| 675 Do you think there are medical treatments to keep you from getting kidney disease or kidney problems? (CHOOSE ONE ONLY) | ◯ | ◯ | ◯ | ◯ | ◯ | ◯ | ◯ |
| 680 If you had kidney problems, how much do you think medical treatments or medications could keep the problems from getting worse? (CHOOSE ONE ONLY) | ◯ | ◯ | ◯ | ◯ | ◯ | ◯ | ◯ |

## Casual Beliefs

| | Not at all | A little bit | Somewhat | Very much | Extremely | DON'T KNOW | REFUSED |
|---|---|---|---|---|---|---|---|
| 690 How much do you think that kidney problems can be caused by high blood pressure? (CHOOSE ONE ONLY) | ◯ | ◯ | ◯ | ◯ | ◯ | ◯ | ◯ |

700 Has your doctor explained to you that kidney problems can be caused by high blood pressure? (CHOOSE ONE ONLY)

◯ Yes
◯ No
◯ Don't Know
◯ Refused

From Saskia: Sanderson et al, 2013
710 How much do you think that people inherit kidney problems through their genes? (CHOOSE ONE ONLY)

◯ Not at all
◯ A little bit
◯ Somewhat
◯ Very much
◯ Extremely
◯ DON'T KNOW
◯ REFUSED

**Now I'd like to ask you some questions about genes and genetics and ways things may be passed down through generations.**
**Knowledge about Genetics: Henneman et al (2004); Sanderson et al (Healthy Subjects WGS study; ENGAGE manuscripts in prep.)**

| | Not at all | A little bit | Somewhat | Very much | Extremely | DON'T KNOW | REFUSED |
|---|---|---|---|---|---|---|---|
| 720 How well do you understand the relationship between genetics and health? (CHOOSE ONE ONLY) | ◯ | ◯ | ◯ | ◯ | ◯ | ◯ | ◯ |

730 Have you ever had a genetic test? That could have been part of this study, or at any other time. (CHOOSE ONE ONLY)

◯ Yes
◯ No
◯ DON'T KNOW
◯ REFUSED

## Beliefs toward genetic testing

| | Not at all | A little bit | Somewhat | Very much | Extremely | DON'T KNOW | REFUSED |
|---|---|---|---|---|---|---|---|
| 740 How well do you understand genetic testing? (CHOOSE ONE ONLY) | ○ | ○ | ○ | ○ | ○ | ○ | ○ |
| 750 In general, do you think it's a good idea to get genetic testing to find out about if you are at risk for getting a common disease like high blood pressure, diabetes or kidney disease? (CHOOSE ONE ONLY) | ○ | ○ | ○ | ○ | ○ | ○ | ○ |
| 760 How much do you think that differences in people's genes affect their risk for these kinds of common diseases? (CHOOSE ONE ONLY) | ○ | ○ | ○ | ○ | ○ | ○ | ○ |
| 770 Do you think African American or Black people are more likely to have diseases like high blood pressure, diabetes and kidney disease than European American or White people? (CHOOSE ONE ONLY) | ○ | ○ | ○ | ○ | ○ | ○ | ○ |
| 780 Do you think these differences in health are because of differences in their genes? (CHOOSE ONE ONLY) | ○ | ○ | ○ | ○ | ○ | ○ | ○ |
| 790 Do you think these differences in health are because of differences in their behaviors or culture? (CHOOSE ONE ONLY) | ○ | ○ | ○ | ○ | ○ | ○ | ○ |
| 800 Do you think these differences in health are because of differences in the amount of stress Black and White people have? (CHOOSE ONE ONLY) | ○ | ○ | ○ | ○ | ○ | ○ | ○ |

| | | | | | | | |
|---|---|---|---|---|---|---|---|
| 805 Do you think these differences in health are because of differences in how much money people have or make? | ◯ | ◯ | ◯ | ◯ | ◯ | ◯ | ◯ |
| 810 Do you think these differences in health are because of differences in their environments, like where they live, their access to care, or pollution? (CHOOSE ONE ONLY) | ◯ | ◯ | ◯ | ◯ | ◯ | ◯ | ◯ |

820 Let's say your doctors thought your risk of getting a disease as a Black/African American person was based on differences in genes, rather than your behavior or your environment. How do you think this would change the way they view you? (CHOOSE ONE ONLY)

◯ It would not change anything
◯ They would have a more negative view of me
◯ They would have a more positive view of me
◯ DON'T KNOW
◯ REFUSED

820a Do you remember getting the APOL1 genetic test? (if NO, remind study participant of their result and practice speakback technique)

◯ Yes
◯ No

**830-920 Knowledge /Beliefs toward APOL1 testing**

**You have been tested (INTERVENTION)/ You have agreed to get tested (CONTROL) for changes in the APOL1 gene that may lead to increased risk of kidney problems or kidney disease.**

| | Not at all | A little bit | Somewhat | Very much | Extremely | DON'T KNOW | REFUSED |
|---|---|---|---|---|---|---|---|
| 830 How well do you understand APOL1 genetic testing? (CHOOSE ONE ONLY) | ◯ | ◯ | ◯ | ◯ | ◯ | ◯ | ◯ |

840 If it were available, how much would you want your children do get tested for changes in the APOL1 gene? (CHOOSE ONE ONLY, THEN IF CONTROL SKIP TO 884)

◯ Not at all
◯ A little bit
◯ Somewhat
◯ Very much
◯ Extremely
◯ NA - No children
◯ DON'T KNOW
◯ REFUSED

Q841-883 ARE ASKED ONLY OF INTERVENTION PARTICIPANTS

841 What was the result of your APOL1 genetic test? (DO NOT READ CHOICES)

☐ Positive (ask what this means and note it)
☐ Negative (ask what this means and note it)
☐ High Risk (ask what this means and note it)
☐ Low Risk (ask what this means and note it)
☐ I have the gene (ask what this means and note it)
☐ I don't have the gene (ask what this means and note it)
☐ Other:
☐ DON'T KNOW
☐ REFUSED

______________________________

Other, specify:

______________________________

842 Do you think that your test result gives you a higher, lower or the same risk of having kidney problems than you had before you had the test?

◯ Higher
◯ Lower
◯ Same
◯ DON'T KNOW
◯ REFUSED

**Decision Regret: Brehaut et al (2002)- Adapted**

**Next, please think about the decision that you made to get your APOL1 genetic test result. Please tell us how strongly you agree or disagree with these statements.**

| | Strongly Agree | Agree | Neither Agree Nor Disagree | Disagree | Strongly Disagree | DON'T KNOW | REFUSED |
|---|---|---|---|---|---|---|---|
| 881 I would go for the same choice (to get my APOL1 genetic test result) if I had to do it over again. (CHOOSE ONE ONLY) | ◯ | ◯ | ◯ | ◯ | ◯ | ◯ | ◯ |
| 882 The choice (to get my APOL1 genetic test result) did me a lot of harm. (CHOOSE ONE ONLY) | ◯ | ◯ | ◯ | ◯ | ◯ | ◯ | ◯ |
| 883 The decision (to get my APOL1 genetic test result) was a wise one. (CHOOSE ONE ONLY) | ◯ | ◯ | ◯ | ◯ | ◯ | ◯ | ◯ |

ONLY 3 AND 12 MONTH: Blue- Psychological Rxn to results) -MICRA (Cella et al, 2002)

884 INTERVENTION: Have you made any changes since you received the results of your APOL1 genetic test last year that had something to do with the study or what you learned from us?
CONTROL: Have you made any changes since you enrolled in the study last year that might have something to do with the study or what you learned from us? (DO NOT READ ANSWER CHOICES)

- ☐ Nothing - I made no changes
- ☐ Tell a friend/relative about the study, the test
- ☐ Be upset/worry
- ☐ Call or go to your doctor
- ☐ Increase amount of physical activity
- ☐ Change diet
- ☐ Drink more water
- ☐ Take blood pressure medications more
- ☐ Stop taking blood pressure medications
- ☐ Reduce/quit drinking
- ☐ Quit smoking
- ☐ Other:
- ☐ DON'T KNOW
- ☐ REFUSED

Other, specify: ______________________________

885 INTERVENTION: Who have you talked to about your results from APOL1 genetic testing?
CONTROL: Who have you talked to about taking part in the study? (CHOOSE ALL) (AFTER RESPONDING TO THIS QUESTION CONTROLS SKIP TO Q886)

- ☐ Doctor or nurse (ASK NEXT QUESTION, IF THEY DID NOT SPEAK TO DOCTOR/NURSE SKIP TO Q886)
- ☐ Family member
- ☐ Friend
- ☐ Other:
- ☐ No one
- ☐ DON'T KNOW
- ☐ REFUSED

Other, specify: ______________________________

885a INTERVENTION: What did your doctor or nurse tell you or suggest that you do?

- ☐ Change meds
- ☐ Take meds
- ☐ Change diet/exercise
- ☐ Nothing
- ☐ Other
- ☐ DON'T KNOW/DON'T REMEMBER
- ☐ REFUSED

Other, specify: ______________________________

885b INTERVENTION: I am satisfied with the way I talked with my doctor or nurse about the APOL1 genetic test.

- ◯ Strongly Agree
- ◯ Agree
- ◯ Neither Agree Nor Disagree
- ◯ DIsagree
- ◯ Strongly Disagree
- ◯ DON'T KNOW
- ◯ REFUSED

886 INTERVENTION: Did your test result change how often you take the blood pressure medications your doctor or nurse has prescribed for you?
CONTROL: Did enrolling in the study change how often you take the blood pressure medications your doctor or nurse has prescribed for you?

◯ Yes
◯ No (SKIP TO Q1000)
◯ DON'T KNOW
◯ REFUSED
◯ I have not been told to take blood pressure medications (SKIP TO Q1000)

887 Do you take your blood pressure medications more often, less often, or the way you took them before? (If pt says they don't take them, ask if they think they would start to) (CHOOSE ONE ONLY- DON'T READ CHOICES)

◯ More often
◯ Less often
◯ The way I took them before.
◯ DON'T KNOW
◯ REFUSED

**FOR CONTROL SKIP TO 1230**

**Now this next set of questions asks specifically about your APOL1 genetic test result and how you felt about it in the last week or 7 days. In the past 7 days...(REPEAT "In the past 7 days" as needed as you go through these questions)**

| | Never | Rarely | Sometimes | Often | DON'T KNOW | REFUSED |
|---|---|---|---|---|---|---|
| 1000 How often have you thought about the results of your APOL1 genetic test? | ◯ | ◯ | ◯ | ◯ | ◯ | ◯ |
| 1010 How ofthen have you felt upset about your results (CHOOSE ONE ONLY) | ◯ | ◯ | ◯ | ◯ | ◯ | ◯ |
| 1020 Felt sad about your results (CHOOSE ONE ONLY) | ◯ | ◯ | ◯ | ◯ | ◯ | ◯ |
| 1030 Felt anxious or nervous about your results (CHOOSE ONE ONLY) | ◯ | ◯ | ◯ | ◯ | ◯ | ◯ |
| 1040 Felt guilty about your results (CHOOSE ONE ONLY) | ◯ | ◯ | ◯ | ◯ | ◯ | ◯ |
| 1050 Felt relieved about your results (CHOOSE ONE ONLY) | ◯ | ◯ | ◯ | ◯ | ◯ | ◯ |
| 1060 Felt happy about your results (CHOOSE ONE ONLY) | ◯ | ◯ | ◯ | ◯ | ◯ | ◯ |
| 1070 Felt a loss of control (CHOOSE ONE ONLY) | ◯ | ◯ | ◯ | ◯ | ◯ | ◯ |

| | | | | | | |
|---|---|---|---|---|---|---|
| 1080 Had problems enjoying life because of your results. (CHOOSE ONE ONLY) | ◯ | ◯ | ◯ | ◯ | ◯ | ◯ |
| 1090 And, in the last week, when thinking about the genetic test result you got back, how often were you worried about your risk of getting kidney problems? (CHOOSE ONE ONLY) | ◯ | ◯ | ◯ | ◯ | ◯ | ◯ |
| 1100 How often have you felt glad you know more about your risk of kidney problems so that you can do something about it? (CHOOSE ONE ONLY) | ◯ | ◯ | ◯ | ◯ | ◯ | ◯ |
| 1110 Felt you weren't sure if your result will affect your risk for kidney problems? (CHOOSE ONE ONLY) | ◯ | ◯ | ◯ | ◯ | ◯ | ◯ |

1120 Felt you weren't sure what your results mean for your children's disease risk? (CHOOSE ONE ONLY)

◯ Never
◯ Rarely
◯ Sometimes
◯ Often
◯ NA - No children
◯ DON'T KNOW
◯ REFUSED

| | Never | Rarely | Sometimes | Often | DON'T KNOW | REFUSED |
|---|---|---|---|---|---|---|
| 1130 Had a hard time deciding whether or not to go to your doctor to check for kidney disease? (CHOOSE ONE ONLY) | ◯ | ◯ | ◯ | ◯ | ◯ | ◯ |
| 1140 And, in the last week, when thinking about the genetic test result you got back how often did you think about how your results may affect your work or your family? (CHOOSE ONE ONLY) | ◯ | ◯ | ◯ | ◯ | ◯ | ◯ |
| 1150 Were you worried your results will cause problems for you getting or keeping your health insurance? (CHOOSE ONE ONLY) | ◯ | ◯ | ◯ | ◯ | ◯ | ◯ |

1160 Did you have trouble  talking about your results with family members?

◯ Never
◯ Rarely
◯ Sometimes
◯ Often
◯ I didn't tell my family (DON'T READ - SKIP TO Q1200)
◯ DON'T KNOW
◯ REFUSED

| | Never | Rarely | Sometimes | Often | DON'T KNOW | REFUSED |
|---|---|---|---|---|---|---|
| 1170 Did you feel your family has been supportive of your decision to get the APOL1 genetic test? | ◯ | ◯ | ◯ | ◯ | ◯ | ◯ |
| 1180 Did you feel satisfied with the way you talked with your family about the APOL1 genetic test? | ◯ | ◯ | ◯ | ◯ | ◯ | ◯ |
| 1190 Did getting the APOL1 genetic test done bring about conflict in your family? | ◯ | ◯ | ◯ | ◯ | ◯ | ◯ |
| 1200 Did you feel regret about getting the APOL1 genetic test done? | ◯ | ◯ | ◯ | ◯ | ◯ | ◯ |

1210 Did you feel guilty about possibly passing on a disease risk to your child(ren)?

◯ Never
◯ Rarely
◯ Sometimes
◯ Often
◯ NA - No children
◯ DON'T KNOW
◯ REFUSED

**Satisfaction with getting results (1 item from Satisfaction with Decision Scale (Holmes-Rovner et al, 1995)**
**Now we'd like you to think about the time you spent learning about the test, deciding to take it and getting the results. Please tell me how much you agree or disagree with the following.**

| | Strongly Agree | Agree | Neither Agree Nor Disagree | Disagree | Strongly Disagree | DON'T KNOW | REFUSED |
|---|---|---|---|---|---|---|---|

| | | | | | | | |
|---|---|---|---|---|---|---|---|
| 1230 I had all the information I needed to make a decision about (INTERVENTION- JOINING THE STUDY, AND GETTING TESTED); (CONTROL- JOINING THE STUDY AND GETTING TESTED LATER). (CHOOSE ONE ONLY) | ○ | ○ | ○ | ○ | ○ | ○ | ○ |
| 1240 The information (INTERVENTION- ABOUT THE STUDY, THE TEST AND THE RESULTS); (CONTROL- ABOUT THE STUDY) was easy to understand. (CHOOSE ONE ONLY) | ○ | ○ | ○ | ○ | ○ | ○ | ○ |

| | Too much | Too little | Just right | DON'T KNOW | REFUSED |
|---|---|---|---|---|---|
| 1250 The amount of information (INTERVENTION- ABOUT THE STUDY, THE TEST AND THE RESULTS); (CONTROL- ABOUT THE STUDY) I got was too much, too little or just right. (CHOOSE ONE ONLY) | ○ | ○ | ○ | ○ | ○ |
| 1260 The amount of time I spent getting the information I needed was too much, too little or just right. (CHOOSE ONE ONLY) | ○ | ○ | ○ | ○ | ○ |

**Depression (PHQ8)**

**Now we'd like to ask you about your mood. Over the past 2 weeks, how often have you been bothered by any of the following problems:**

| | Not at all | Several days | More than half the days | Nearly everyday | DON'T KNOW | REFUSED |
|---|---|---|---|---|---|---|
| 1270 Little interest or pleasure in doing things (CHOOSE ONE ONLY) | ○ | ○ | ○ | ○ | ○ | ○ |
| 1280 How often have you been bothered by: Feeling down, depressed, or hopeless (CHOOSE ONE ONLY) | ○ | ○ | ○ | ○ | ○ | ○ |
| 1290 How often have you been bothered by: Trouble falling or staying asleep, or sleeping too much (CHOOSE ONE ONLY) | ○ | ○ | ○ | ○ | ○ | ○ |

| | | | | | | |
|---|---|---|---|---|---|---|
| 1300 How often have you been bothered by: Feeling tired or having little energy (CHOOSE ONE ONLY) | ○ | ○ | ○ | ○ | ○ | ○ |
| 1310 Over the past 2 weeks, how often have you been bothered by: Poor appetite or overeating (CHOOSE ONE ONLY) | ○ | ○ | ○ | ○ | ○ | ○ |
| 1320 How often have you been bothered by: Feeling bad about yourself, or that you are a failure or have let yourself or your family down (CHOOSE ONE ONLY) | ○ | ○ | ○ | ○ | ○ | ○ |
| 1330 Over the past 2 weeks, how often have you been bothered by: Trouble concentrating on things, such as reading the newspaper or watching television (CHOOSE ONE ONLY) | ○ | ○ | ○ | ○ | ○ | ○ |
| 1340 How often have you been bothered by: Moving or speaking so slowly that other people could have noticed-- or the opposite - being so fidgety or restless that you have been moving around a lot more than usual. (CHOOSE ONE ONLY) | ○ | ○ | ○ | ○ | ○ | ○ |

**GAD-2 (Generalized Anxiety)**

**Over the last two weeks, how often have you been bothered by the following problems?**

| | Not at all | Several days | More than half the days | Nearly every day | DON'T KNOW | REFUSED |
|---|---|---|---|---|---|---|
| 1350 Feeling nervous, anxious or on edge (CHOOSE ONE ONLY) | ○ | ○ | ○ | ○ | ○ | ○ |
| 1360 Not being able to sleep or control worrying? (CHOOSE ONE ONLY) | ○ | ○ | ○ | ○ | ○ | ○ |

## 4-Item Perceived Stress Scale - Cohen, et al. 1988

| | Never | Almost Never | Sometimes | Fairly Often | Very Often | DON'T KNOW | REFUSED |
|---|---|---|---|---|---|---|---|
| 1370 In the last month, how often have you felt that you were not able to control the important things in your life? (CHOOSE ONE ONLY) | ◯ | ◯ | ◯ | ◯ | ◯ | ◯ | ◯ |
| 1380 In the last month, how often have you felt confident about your ability to handle your personal problems? (CHOOSE ONE ONLY) | ◯ | ◯ | ◯ | ◯ | ◯ | ◯ | ◯ |
| 1390 In the last month, how often have you felt that things were going your way? (CHOOSE ONE ONLY) | ◯ | ◯ | ◯ | ◯ | ◯ | ◯ | ◯ |
| 1400 In the last month, how often have you felt difficulties were piling up so high that you could not overcome them? (CHOOSE ONE ONLY) | ◯ | ◯ | ◯ | ◯ | ◯ | ◯ | ◯ |

Lifestyle- Diet/Exercise: Adapted from NHANES 2013

1530 Now I'd like to ask you some questions about your lifestyle. During the past 7 days, how much physical activity (like walking) or exercise did you do? (CHOOSE ONE ONLY)

◯ Less than an hour
◯ 1 to 1 ½ hours
◯ 1 ½ to 2 hours
◯ More than 2 hours
◯ DON'T KNOW
◯ REFUSED

1540 Have you become more physically active or exercised more in the past 6 months? (CHOOSE ONE ONLY)

◯ Yes
◯ No
◯ DON'T KNOW
◯ REFUSED

1550 In general, do you eat a healthy diet? (CHOOSE ONE ONLY)

◯ Not at all
◯ A little bit
◯ Somewhat
◯ Very much
◯ Extremely
◯ DON'T KNOW
◯ REFUSED

1560 Have you made changes to your diet in the past 6 months? (CHOOSE ONE ONLY)

◯ Yes, my diet is healthier
◯ Yes, my diet is less healthy
◯ No
◯ DON'T KNOW
◯ REFUSED

1570 Now we'd like to ask you about some of your other health habits: In the past month, did you smoke or chew tobacco? (CHOOSE ONE ONLY)

◯ Yes
◯ No (GO TO Q1600)
◯ DON'T KNOW
◯ REFUSED

1580 How many cigarettes or cigars on average do you smoke each day? (CHOOSE ONE ONLY)

______________________________
(If < 1, write '0'. If participant doesn't know, write "888")

1590 Do you currently want to quit smoking?

◯ Yes
◯ No
◯ DON'T KNOW
◯ REFUSED

1600 How often do you have a drink containing alcohol? (CHOOSE ONE ONLY)

◯ Never (GO TO Q1800)
◯ Monthly or less
◯ 2 to 4 times a month
◯ 2 to 3 times a week
◯ 4 or more times a week
◯ DON'T KNOW
◯ REFUSED

1610 How many drinks containing alcohol do you have on a typical day when you are drinking? (CHOOSE ONE ONLY - DON'T READ OPTIONS)

◯ None
◯ 1 or 2
◯ 3 or 4
◯ 5 or 6
◯ 7 to 9
◯ 10 or more
◯ DON'T KNOW
◯ REFUSED

1620 How often do you have 6 or more drinks on one occasion? (CHOOSE ONE ONLY)

◯ Never
◯ Less than monthly
◯ Monthly
◯ Weekly
◯ Daily
◯ DON'T KNOW
◯ REFUSED

Finally, we'd like to ask you some questions about your medical care.

Health Care Utilization

1800 Do you currently have a person you consider your primary care doctor or primary care nurse? (CHOOSE ONE ONLY)

◯ Yes
◯ No
◯ DON'T KNOW
◯ REFUSED

1810 In the last 6 months, how many times did you see any primary care doctor? (CHOOSE ONE ONLY)

______________________________
(For Don't know type: 888; For Refused, type:999)

1820 On average, how long does it take you to get to your doctor's office from your home? (Enter number of MINUTES)

______________________________
(For Don't know type: 888; For Refused, type:999)

1830 On average, how much time do you spend at your primary care doctor's office? Please include both the time you wait to see your doctor and the time the doctor spends with you. (Enter number of MINUTES)

______________________________
(For Don't know type: 888; For Refused, type:999)

1840 What form of transportation do you usually use to get to your primary care doctor's office? (CHOOSE ONE ONLY)

◯ Public Transportation
◯ Ambulette/ Access-a-Ride
◯ Friend or Family drives me
◯ Taxi
◯ Walking
◯ Other
◯ I drive myself
◯ DON'T KNOW
◯ REFUSED

Other, specify:

______________________________

1850 In the last 6 months, how many times did you visit specialist doctors? That is, how many visits did you make to doctors other than your primary care doctor such as to heart or kidney doctors? (CHOOSE ONE ONLY)

______________________________

(For Don't know type: 888; For Refused, type:999)

1860 In the last 6 months, how many times did you go to an emergency room? (CHOOSE ONE ONLY)

______________________________

(For Don't know type: 888; For Refused, type:999)

1870 In the last 6 months, how many times were you admitted to the hospital? That is, how many times did you spend more than 24 hours in the hospital and not just in the emergency room? (CHOOSE ONE ONLY)

______________________________

(For Don't know type: 888; For Refused, type:999)

1880 In the past six months, how many times did you see a mental health provider, such as a psychologist, psychiatrist, or social worker? (CHOOSE ONE ONLY)

______________________________

(For Don't know type: 888; For Refused, type:999)

Now we'd like to ask you a few questions about you!

1950 What kind of health insurance, if any, do you have right now? (CHOOSE ALL APPLY)

☐ No Insurance/Sliding Scale
☐ Medicaid (SKIP TO Q1970)
☐ Medicare (SKIP TO Q1970)
☐ Private Health Insurance (SKIP TO Q1970)
☐ Military Health care/VA (SKIP TO Q1970)
☐ Other Insurance (specify) (SKIP TO Q1970)
☐ DON'T KNOW
☐ REFUSED

Other, specify:

______________________________

1960 How long has it been since you last had health coverage? (CHOOSE ONE ONLY)

◯ 6 months or less
◯ More than 6 months, up to and including 1 year
◯ More than 1 year
◯ DON'T KNOW
◯ REFUSED

1970 Are your prescription medications covered (at least partially) by some kind of health insurance? (CHOOSE ONE ONLY)

◯ Yes
◯ No
◯ DON'T KNOW
◯ REFUSED

Thank you, you have finished the survey!
