# Supplementary material for: Effects of Testing and Disclosing Ancestry-Specific Genetic Risk for Kidney Failure on Patients and Health Care Professionals: A Randomized Clinical Trial
Source: JAMA Netw Open. 2022 Mar 4;5(3):e221048. doi: 10.1001/jamanetworkopen.2022.1048 (PMC8897752; doi:10.1001/jamanetworkopen.2022.1048)
Supplement: Supplement 2. — Data Sharing Statement [file jamanetwopen-e221048-s002.pdf]

Data Sharing Statement

Nadkarni. Effects of Testing and Disclosing Ancestry-Specific Genetic Risk for Kidney Failure on Patients and Health Care Professionals. *JAMA Netw Open*. Published March 04, 2022. doi:10.1001/jamanetworkopen.2022.1048

Data
Data available: Yes
Data types: Deidentified participant data
How to access data: carol.horowitz@mountsinai.org
When available: With publication

Supporting Documents
Document types: Other (please specify)
Additional Information: Trial protocol and surveys
How to access documents: supplementary appendix
When available: With publication

Additional Information
Who can access the data: anyone requesting the data
Types of analyses: any purpose
Mechanisms of data availability: signed data access agreement
Any additional restrictions: None
